# An ecosystem service approach to the study of vineyard landscapes in the context of climate change: a review

**DOI:** 10.1007/s11625-022-01223-x

**Published:** 2022-09-17

**Authors:** Sebastian Candiago, Klara Johanna Winkler, Valentina Giombini, Carlo Giupponi, Lukas Egarter Vigl

**Affiliations:** 1 Institute for Alpine Environment, Eurac Research, Viale Druso 1, 39100 Bozen/Bolzano, Italy; 2 Department of Economics, Ca’ Foscari University of Venice, S. Giobbe 873, 30121 Venice, Italy; 3 McGill University, Macdonald Campus, 21,111 Lakeshore Drive, Ste-Anne-de-Bellevue, QC H9X 3V9 Canada

**Keywords:** Viticulture, Agricultural system, Socio-ecological system, Ecological condition, Global warming, Adaptation

## Abstract

Vineyard landscapes significantly contribute to the economy, identity, culture, and biodiversity of many regions worldwide. Climate change, however, is increasingly threatening the resilience of vineyard landscapes and of their ecological conditions, undermining the provision of multiple ecosystem services. Previous research has often focused on climate change impacts, ecosystem conditions and ecosystem services without systematically reviewing how they have been studied in the literature on viticulture. Here, we systematically review the literature on vineyard landscapes to identify how ecosystem conditions and services have been investigated, and whether an integrative approach to investigate the effects of climate change was adopted. Our results indicate that there are still very few studies that explicitly address multiple ecosystem conditions and services together. Only 28 and 18% of the reviewed studies considered more than two ecosystem conditions or services, respectively. Moreover, while more than 97% of the relationships between ecosystem conditions and services studied were addressing provisioning and regulating services, only 3% examined cultural services. Finally, this review found that there is a lack of integrative studies that address simultaneously the relationships between ecosystem condition, ecosystem services and climate change (only 15 out of 112 studies). To overcome these gaps and to better understand the functioning of vineyard socio-ecological systems under climate change, multidisciplinary, integrative, and comprehensive approaches should be adopted by future studies. A holistic understanding of vineyard landscapes will indeed be crucial to support researchers and decision makers in developing sustainable adaptation strategies that enhance the ecological condition of vineyards and ensure the provision of multiple ecosystem services under future climate scenarios.

**Supplementary Information:**

The online version contains supplementary material available at 10.1007/s11625-022-01223-x.

## Introduction

Vineyard landscapes (VLs) are important agroecosystems that provide multiple economic, ecological, and cultural benefits, or ecosystem services, to society (Table 1). Due to the economic value of wine grapes, viticulture and winemaking shape the socio-economic system of many winegrowing regions worldwide (Fraga et al. 2012; Azorín and García 2020). The mosaic of land uses within VLs, including croplands, forests, shrublands and riparian areas, also supports the biodiversity and ecosystem functioning of these regions (Viers et al. 2013; Winkler and Nicholas 2016; Winter et al. 2018). In addition to these economic and ecological qualities, VLs are also often defined as cultural landscapes that provide a variety of intangible benefits to residents and visitors alike (Winkler and Nicholas 2016).

**Table 1:** Definition of the key concepts used in this review

Term	Acronym	Definition
Agroecosystem	–	Agricultural ecosystems including biophysical and human components and their interactions (Garbach et al. 2014)
Ecosystem conditions	EC	The physical, chemical, and biological characteristics or qualities of an ecosystem at a particular point in time (Maes et al. 2018)
Ecosystem services	ES	Contributions of ecosystems to human benefits obtained from economic, social, cultural and other human activities (SEEA-EEA 2012)
Climate change	CC	A change in climate that is attributed directly or indirectly to human activity that alters the composition of the global atmosphere and that is in addition to natural climate variability observed over comparable time periods (UNFCCC 1992)
Vineyard landscape	VL	A mosaic of farmers' fields, semi-natural habitats, human infrastructure (e.g., roads) and occasional natural habitats (Marshall 2004), where the major agricultural activity is viticulture
Links	–	Relationship between two components of a system as they are studied in the literature (Falardeau and Bennett 2019)

The range of ecosystem services provided by VLs is largely determined by the physical, chemical, and biological conditions, or quality, (i.e., ecosystem condition) of each agroecosystem at a particular point in time (Maes et al. 2016, 2018; Kokkoris et al. 2018). The relationship between ecosystem conditions and services is particularly evident in traditional VLs, which have developed over time as a result of a close relationship between local environmental conditions and human activities. However, different drivers of change can exert multiple pressures on ecosystem conditions and can have direct and indirect impacts on the related ecosystem services (Maes et al. 2018). In particular, climate change effects such as higher temperatures and altered precipitation regimes are already posing significant challenges to the integrity and condition of many VLs and are, thus, altering the capacity of these systems to deliver a variety of provisioning, regulating, and cultural ecosystem services (Hannah et al. 2013; Bindi and Nunes 2016; Maes et al. 2018). Moreover, changes in climatic conditions are also reflected in more complex human–nature interactions related to land conversions, as new land at the cooler end of the vine suitability spectrum is becoming increasingly available or as established vineyards are being abandoned (Vigl et al. 2018).

Over the past years, the analysis of the relationships between ecosystem conditions, ecosystem services, and climate change in VLs has received attention in literature on viticulture. Studies have looked, for example, at the effects of climate change on those conditions and services important for food production, such as yield, plant growth, and soil fertility (Tancoigne et al. 2014; Winkler et al. 2017; Nieto-Romero et al. 2014). Winkler et al. (2017), in their review paper, were among the first to introduce the importance of considering the multiple ecosystem functions and services provided by VLs. They found that viticulture research mainly addressed provisioning and regulating ecosystem services, looking at VLs as agrarian landscapes. Studies that address multiple ecosystem conditions or services simultaneously (i.e., comprehensive research) instead also recognize and highlight other important co-benefits (Power 2010; Maes et al. 2018). Indeed, researchers have recently started to examine more systematically the full array of ecosystem services provided by VLs, also including socio-cultural services such as heritage, identity, and esthetics (Sottini et al. 2019; Garcia et al. 2018).

To face the complexities of an increasingly interconnected world where disciplinary or sectoral approaches have had limited success, it is necessary to develop and apply holistic thinking (Wezel et al. 2020). In fact, the co-creation of knowledge from different disciplines (i.e., multidisciplinarity), has been listed as one of the main elements that can support the development of transformative change pathways towards sustainable food and agricultural systems (FAO 2019). Analyzing the multiple ecosystem conditions and services provided by VLs embracing a multidisciplinary perspective can provide opportunities for the sustainable management of agroecosystems that cannot be obtained by adopting single discipline approaches (Stark 1995).

In recent decades, ecosystem services research has showed the importance of considering both ecosystem conditions and anthropogenic pressures to understand how the benefits of nature are delivered to society (Maes et al. 2018). Adopting an ecosystem service approach in the study of agroecosystems fosters research that disentangles the relationships among different components of a socio-ecological system, i.e., integrative research (Falardeau and Bennett 2019; Liu et al. 2014). Consequently, adopting an integrative approach enables one to study how an ecosystem condition is affected by climate change and which are the related consequences on the provision of an ecosystem service (Falardeau and Bennett 2019; Kluger et al. 2020). The results of such integrative research would allow the increase of knowledge on the components of these socio-ecological systems and their relationships, providing the insights and recommendations needed to support decision makers in developing strategies that enhance the provision of ecosystem services while addressing the potential negative effects of drivers of change (Maes et al. 2018; Falardeau and Bennett 2019). This is particularly important in view of the management of VLs under new climate scenarios.

Decision makers working to ensure the resilience of VLs under climate change require timely and thorough knowledge on the relationships between climate change, ecosystems, and desired ecosystem services. In the past, however, there has not always been the interest to explore all these relationships, and there is the need to target research to explore missing and understudied linkages to avoid maladaptation or unintended consequences to policy interventions. Understanding which relationships among VL components have been explored so far by academia is enabled by having a systematic knowledge on the studies that have been carried out on VLs. Conducting a systematic review can produce this knowledge, as it would systematically search for, appraise, and synthetize available research on selected components of VLs, following specific guidelines and ensuring rigorousness and full replicability (Grant and Booth 2009). This would allow one to identify the links between those components for which more research is needed, and to fill important research gaps in the literature. To our knowledge, however, no systematic reviews have so far reviewed how the relationships between climate change and multiple ecosystem conditions and services in VLs have been studied in the literature.

In this study, we carry out a systematic literature review to examine how ecosystem conditions in VLs are studied in relation to the provision of ecosystem services, and how both are investigated in the context of climate change. We provide indications to researchers on which relationships among climate change, ecosystem conditions and services in VLs have been studied, and to what extent. Our objectives are:

(i) To identify the main spatiotemporal patterns and the disciplines found in the literature on ecosystem conditions, ecosystem services, and climate change in VLs.
(ii) To analyze how the relationships between ecosystems conditions and ecosystem services in VLs are studied.
(iii) To understand how climate change is considered in the study of the ecosystem conditions and ecosystem services in VLs.

## Materials and methods

### Literature search and selection

We identified peer-reviewed publications from the online databases Scopus and Web of Science following the steps of the Preferred Reporting Items for Systematic reviews and Meta-Analyses (PRISMA) methodology (Moher et al. 2009) (supplementary figure S1), building on the search structure used also by Falardeau and Bennett (2019). We specifically looked for research papers dealing with VLs that investigated links between ecosystem conditions, ecosystem services, and climate change variables. The set of terms used to search for relevant publications included (*a set of terms relevant to winegrowing*) AND (*terms connected with climate change*) AND (*terms related to ecosystem conditions* OR *terms related to ecosystem services*). To thoroughly search for relevant literature regarding climate change, ecosystem conditions, and ecosystem services, we performed several queries to tailor our search on each of the ecosystem conditions and services considered in the context of climate change. We started by running a query to find articles published on the effects of climate change on the ecosystem conditions in VLs, using the European framework proposed for the Mapping and Assessment of Ecosystems and their Services (Maes et al. 2018) to define our search terms related to ecosystem conditions. Then, we ran a set of twenty-eight tailored queries, one for each ecosystem service class included in this review, to include literature on those ecosystem services, viticulture, and the effects of climate change (see tables S1 and S2). The ecosystem service classes and the related synonyms included in each of these queries were based on the work by Winkler et al. (2017). Finally, to intercept all relevant literature that studied ecosystem services in VLs, we also ran a general query without specifying any ecosystem service classes nor terms connected to climate change.

We combined the results of our search strings using the R-package “bibliometrics” (Aria and Cuccurullo 2017; R Core Team 2020), (supplementary tables S1 and S2). Our search was conducted on March 25, 2020, obtaining over 1,600 potentially relevant articles. After removing duplicates (*n* = 986), we screened the titles, keywords and abstracts of 661 articles by applying a set of inclusion and exclusion criteria (supplementary table S3). To screen the content of the papers, we combined manual and automatic techniques using the QCRI Rayyan software (Ouzzani et al. 2016). Rayyan is a free systematic review software that facilitates the initial screening of abstracts and titles using a process of semi-automation (Harrison et al. 2020). The software uses machine learning to increase the speed of the screening process, using the inclusion/exclusion decisions made by the user on a sample of papers to score the likelihood that studies awaiting screening will be included. As suggested by the methodology used in previous reviews, we manually assessed 10% of the records uploaded in Rayyan to train the machine learning algorithm that automatically screened the remaining studies (Garrick et al. 2019). After applying the trained algorithm, we conducted a manual validation of the results. For this purpose, we selected an additional 10% of the automatically classified articles and checked them, paying attention to keeping training and validation datasets separate. We found a high correspondence (> 90%) between the results of the automatic classification and the results of the manual quality control. After the screening process, we assessed the full texts of 208 papers and ultimately retained 112 articles in our review.

### Relationships between ecosystem conditions, ecosystem services and climate change addressed by the literature

To review with an integrative perspective how ecosystem conditions, ecosystem services, and climate change variables have been studied in the literature, we counted each time the relationship (i.e., a link) between climate change, ecosystem conditions, and ecosystem services was addressed in a reviewed paper (Menegon et al. 2018; Carter et al. 1994; Falardeau and Bennett 2019). We considered as a “link” any relationship between system components which was investigated with qualitative or quantitative methods, even if it was found to be a non-significant correlation. The aim of the present study is indeed to understand how research on VLs has been conducted by academia until now, and not to investigate the biophysical processes occurring in VLs.

**Fig. 1:**
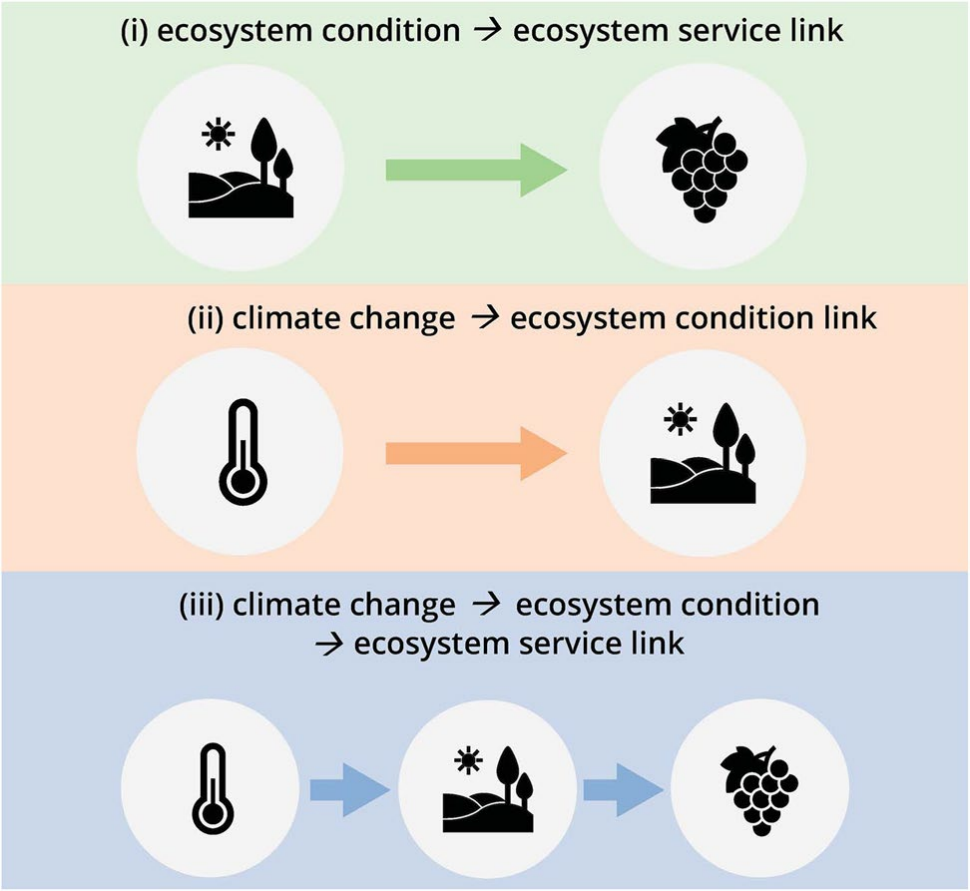
Representation of the three link types looked for in the review of the papers. The main components of VLs that we considered were ecosystem conditions, ecosystem services and climate change

The three types of links that we considered were (Fig. 1):

(i) *ecosystem condition* *ecosystem service*
(ii) *climate change* *ecosystem condition*
(iii) *climate change* *ecosystem condition* *ecosystem service*

For example, when Fraga et al. (2019) studied the influence of phenology on the amount of grapes produced (crop production), the authors described an *ecosystem condition* *ecosystem service* link, specifically a *phenology* *crop production* link. In the same way, if a paper assessed an influence of temperature on phenology, then it described a *climate change* *ecosystem condition* link, and thus, we recorded the link *temperature* *phenology*. Lastly, if a paper described the influence of temperature on phenology and the related effects of phenology on crop production, then we recorded the integrative link *temperature* *phenology* *crop production* (*climate change* *ecosystem condition* *ecosystem service*). These three types of links are highly interrelated and interdependent. For this reason, we analyzed them jointly by following the structure of the framework developed by the European mapping and assessment of ecosystems and their services (Maes et al. 2018). This enabled us to identify which relationships amongst ecosystem conditions, ecosystem services, and climate change were more or less frequently studied.

### Extracting information from the articles

We retrieved information from the articles based on a set of structured questions (Table 2). For our first research objective, we started by analyzing the context, focus and disciplines of each paper (Q1–Q7). For our second research objective, we retrieved information on how and which ecosystem conditions and ecosystem services were studied in the articles (Q8–Q10). For our third research objective, we investigated how the influence of climate change on VLs was investigated (Q11–Q12).

**Table 2:** Questions used to extract relevant information from the reviewed literature (*n* = 112)

Theme	Question	Id	Objective
Context	What is the year of publication and type of article?	Q1	(i)
	What is the spatial scale of assessment?	Q2	(i)
	What is the temporal scale of assessment?	Q3	(i)
Focus	Does the paper focus only on *ecosystem conditions* *ecosystem services* links?	Q4	(ii)
	Does the paper include *climate change* *ecosystem conditions* or *climate change* *ecosystem conditions* *ecosystem services* links?	Q5	(iii)
Disciplines	Which disciplines are involved in the study of ecosystem conditions and services?	Q6	(i), (ii)
	Which disciplines are involved in the study of climate change variables?	Q7	(i), (iii)
Ecosystem conditions and services	Which are the ecosystem conditions considered?	Q8	(ii)
	Which are the ecosystem services considered?	Q9	(ii)
	Which *ecosystem conditions* *ecosystem services* links are studied?	Q10	(ii)
Climate change	Which are the climate change variables considered?	Q11	(iii)
	Which *climate change* *ecosystem conditions* or *climate change* *ecosystem conditions* *ecosystem services* links are studied?	Q12	(iii)

Options for answering the questions are explained in supplementary table S4

## Results

### Context and focus of the articles

**Fig. 2:**
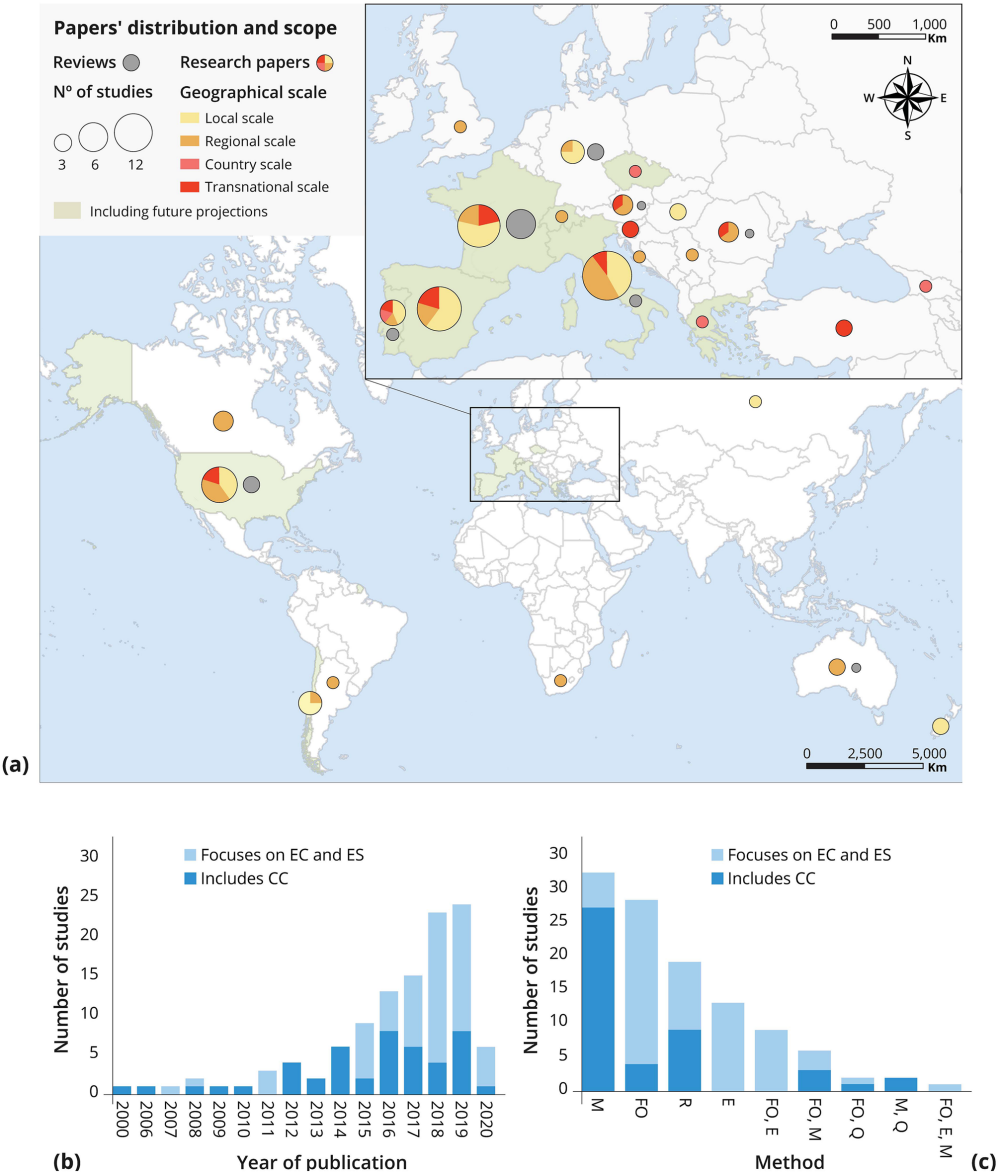
Key features of the 112 articles included in our literature review: **a** spatial distribution of the analyzed articles. Research papers are classified based on their geographic scale and the inclusion of future projections in their analysis. Criteria for spatial and temporal classification are provided in supplementary table S4. Review papers are classified separately (gray circles) based on their geographic location, defined using the affiliation of their first author; **b** bar chart for each year of publication, classified by the thematic focus of the articles; **c** bar chart representing the methodology used (M = model, FO = field observation, R = literature review, E = field experiment, Q = questionnaire) to study the links between ecosystem conditions (EC), ecosystem services (ES), and climate change (CC), classified by the thematic focus of the articles

The spatial distribution of the reviewed studies corresponded to the locations of the world’s main viticulture regions. Most articles have been published over the last decade and looked at European VLs (74%, Fig. 2a). All the studies we found were only published after 2000, with 85% of them published since 2013 (Fig. 2b). Nevertheless, most papers had a regional or local focus, while only 10% of the cases had a transnational or national scope. Regarding the temporal perspective, future scenarios were included in nine out of the 112 investigated articles, while the other articles were based on data from past and present observations.

We found that 60% of our papers focused only on *ecosystem conditions* *ecosystem services* links, while 40% included *climate change* *ecosystem conditions* or *climate change* *ecosystem conditions* *ecosystem services* links. Of those studies that focused on *ecosystem conditions* *ecosystem services* links, 63% were published after 2015. The number of studies that included climate change-related links remained consistent over the reviewed time period (Fig. 2b). Most of the articles that used a modeling approach considered climate change variables (84%, Fig. 2c), whilst the articles using field observations or experiments mainly focused on *ecosystem conditions* *ecosystem services* links (86 and 100% of the articles, respectively). Literature review approaches were used by both those studies that focused on ecosystem conditions and services, and those that included climate change variables. Finally, 70% of the articles that adopted more than one method were investigating *ecosystem conditions* *ecosystem services* links. The combination of models and questionnaires was used only in papers that included climate change-related links.

### Ecosystem conditions and ecosystem services

**Fig. 3:**
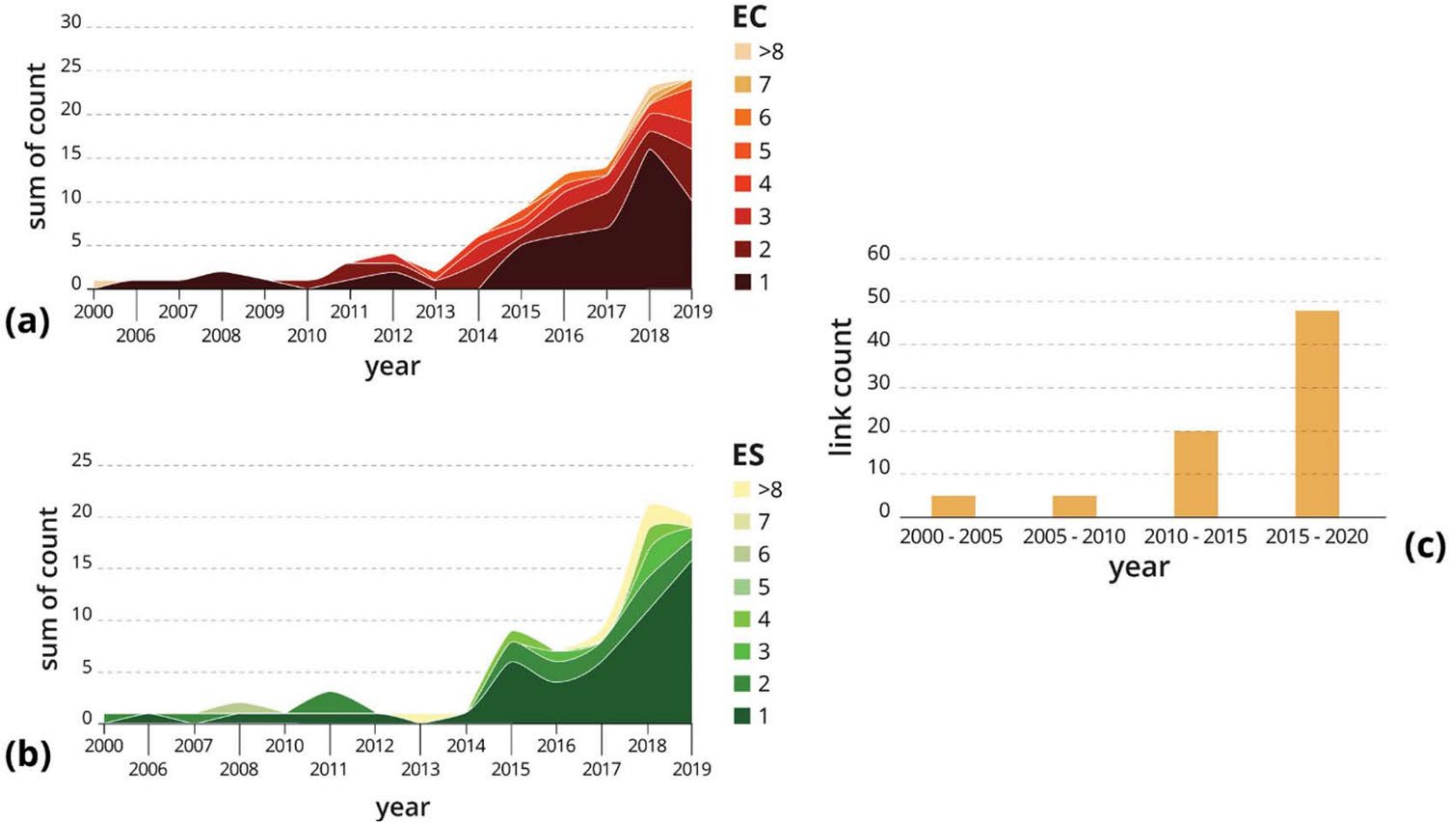
**a** Number of ecosystem conditions considered together in our sample of papers over time; **b** number of ecosystem services considered in our sample of papers over time; **c** the number of *climate change* *ecosystem conditions* *ecosystem services* links considered over time

**Fig. 4:**
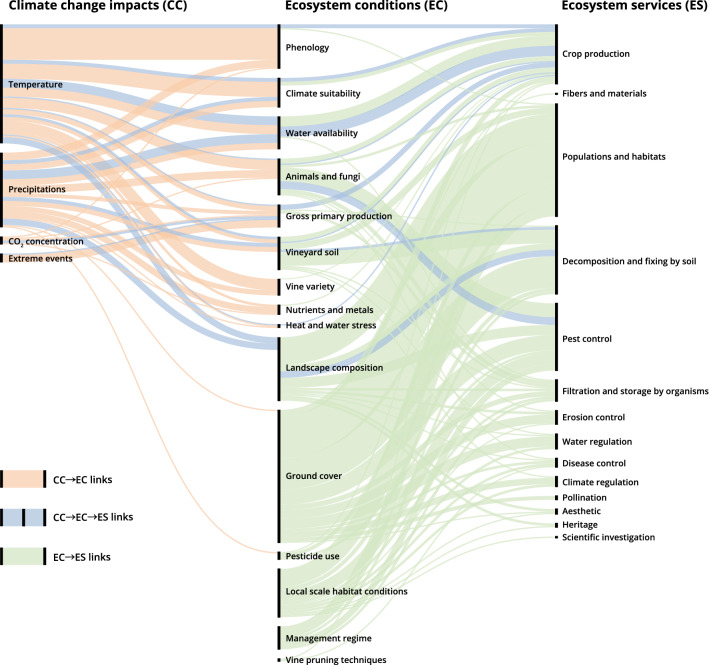
Sankey diagram representing the *ecosystem conditions* *ecosystem services* links, *climate change* *ecosystem conditions* links, and *climate change* *ecosystem conditions* *ecosystem services* links retrieved in our review. The thickness of the lines is proportional to the total number of links. The percentages of how much the single links’ components and their relationships were studied are reported in supplementary figure S4 and table S7)

In our review, we found a total of 276 *ecosystem conditions* *ecosystem services* links in 76 papers. The most studied ecosystem conditions included in this link type were ground cover conditions (38%), landscape composition (16%), local habitat conditions (14%), vineyard soil conditions (8%), presence of animals or fungi (8%), management regime (7%) and water availability (3%) (Fig. 4). Most of the articles that included *ecosystem conditions* *ecosystem services* links considered only one single ecosystem condition (57%), (supplementary figure S2a). The study by Winkler et al. (2017), for example, was one of the few cases that analyzed how multiple ecosystem conditions, such as landscape composition, vineyard soil, canopy management strategies, and presence of natural enemies, affect the provision of multiple ecosystem services in VLs. The most studied ecosystem services were those related to the maintenance of nursery beneficial populations and habitats (32%), pest control (17%), decomposition and fixing processes and their effects on soil (17%), crop production (9%), and filtration and storage by organisms (6%). When looking at the temporal patterns in the publication of the reviewed articles, we found that although only a limited number of papers studied multiple ecosystem conditions and services (Fig. 3a, b), an increasing number of linkages can be observed.

As found for ecosystem conditions, also ecosystem services were considered mainly individually. In fact, 62% of the papers that included an *ecosystem conditions* *ecosystem service* link considered only one ecosystem service, and 19% considered two. Studies that considered three or more ecosystem services were less than 20%. For example, Viers et al. (2013) included 11 different ecosystem services when reviewing potential benefits provided by VLs (supplementary figure S2b).

We found that the *ecosystem conditions* *ecosystem services* links focused mainly on a specific set of ecosystem conditions that was particularly studied in relation to the regulating and provisioning ecosystem services that are important for wine grape production. For example, ground cover conditions in the inter-row spaces of vineyards have been extensively studied, especially for their potential to limit weed establishment and to maintain populations and habitats of species that prey on pests (16% of the links), such as in Hoffmann et al. (2017). Ground cover was also studied regarding its effect on other services, showing that it is useful for increasing the decomposition and fixing processes of the soil, regulating the water cycle, and protecting the soil against erosion, (15% of the links), (Nistor et al. 2018; Shields et al. 2016; Winter et al. 2018).

Landscape composition was studied in relation to the provision of many services. The presence of semi-natural areas near vineyards and landcover heterogeneity at the landscape scale were studied in relation to the capacity to provide habitats and increase the populations of species that are beneficial for vintners and for the biological control of pests (11% of the links). For example, Rusch et al. (2016) found that the presence of diverse natural habitats enhanced ground beetle species turnover, supporting more heterogeneous insect communities in simple landscapes. The same author analyzed the pest control of grape berry moths in Bordeaux vineyards, concluding that landscape heterogeneity was the main variable affecting the biological control of these insects (Rusch et al. 2017). Local habitat conditions were studied based on specific elements, including the presence of solitary trees or green infrastructure such as hedgerows, that can provide habitat for beneficial animals, (5% of the links), e.g., in Polyakov et al. (2019), Rosas-Ramos et al. (2019). We found that habitats characteristic of VLs, such as stone walls and hedgerows, were also investigated for the provision of cultural services such as those related to esthetic perceptions or cultural heritage (Assandri et al. 2018). The presence of animals or fungi was studied in terms of pest control (4% of the links). For instance, we found multiple studies that examined the activity of arthropods, birds and bats in vineyards and their role as predators against pests such as grape berry moths, e.g., Thiéry et al. (2018). In addition, we found that specific organisms, such as arbuscular mycorrhizae, were considered for their benefits to VLs, e.g., alleviation of grapevine water stress (Trouvelot et al. 2015).

Vineyard soil conditions were analyzed considering ecosystem services related to decomposition and fixing processes (4% of the links), for example studying the fraction of organic carbon in the soil, which influences carbon sequestration (Nistor et al. 2018; Novara et al. 2018). Vineyard management regimes were mostly studied by analyzing the effect of organic practices on the enhancement of beneficial populations, habitats, and pest control effects (5% of the links), e.g., in Muneret et al. (2019). Water availability was studied in relation to grape production (3% of the links), as Bernardo et al. (2018) and Schultz (2016) showed that this is an important condition for the formation and development of grape berries.

### Climate change

We found 122 *climate change* *ecosystem conditions* links stemming from 46 papers. The most studied climate change variables in these links were temperature (58%), followed by precipitation (34%), extreme events (5%) and CO_2_ concentration (3%) (Fig. 4). In 50% of the cases, articles that included *climate change* *ecosystem conditions* links considered only one ecosystem condition (supplementary figure S2d). In 46% of the cases, two or more climate change variables, especially temperature and precipitation, were included (Figure S2c). The most studied ecosystem conditions were phenology (26%), climatic suitability for viticulture (14%), the presence of animals or fungi (12%), and gross primary production (11%).

In 20% of *climate change* *ecosystem conditions* links, temperature was studied either in relation to the advancement of the phenological stages of grapevines, like in Fraga et al. (2016), or based on the recorded harvest dates as a proxy for vine phenology for premium wine estates, e.g., Carlo et al. (2019). Temperature was also shown to not only influence specific plant-dependent processes but also the overall climatic suitability for viticulture in many areas (10% of the *climate change* *ecosystem conditions* link). For example, increasing temperatures are threatening grape production in many traditional grape growing regions and increasing the suitability of new areas for viticulture (Fraga et al. 2016). The influence of temperature in the regulation of the water cycle was reflected in the number of links (7%) retrieved from studies that highlighted how the increase in temperature will decrease the water reservoirs upon which some VLs depend, e.g., in Castex et al. (2015), and increase evapotranspiration, leading to water deficits and changes in several vine yield parameters, e.g., in Leeuwen et al. (2019). Temperature was moreover investigated due to its influence on animals and fungi present in VLs (7% of the links), as it was shown to possibly increase pest activity in viticultural areas by creating more suitable climatic conditions (e.g., Nesbitt et al. (2016), Rayne and Forest (2016)). Precipitation was studied primarily in terms of water availability in vineyards and climatic suitability for viticulture (8% of the links). The decrease of water availability was shown to negatively affect the overall quantity of water for the physiological activities of the vines (Lazoglou et al. 2018). Increased moisture due to higher precipitation was related to the presence of fungi in vineyards and to the risk of fungal pathogen outbreaks and disease pressure (Neethling et al. 2019). Precipitation was also found to affect phenology (5% of the links), such as in Ramos et al. (2018). Extreme events such as hail and heavy storms were considered only in 5% of the *climate change* *ecosystem conditions* links, even if these events can heavily influence the gross primary productivity of vines by damaging plants in sensitive phenological phases such as budburst, as showed by Nesbitt et al. (2016) and Neethling et al. (2019). Finally, the increase in CO_2_ concentration was primarily studied in relation to the presence of animals and fungi and to the changes in phenology and gross primary production (2% of the links). For example, Schultz (2016) reported that an increase in CO_2_ concentration can be beneficial for the biomass production of vines but could also lead to an increase in the activity of insects, which will result in more damage to plants. A higher CO_2_ concentration in combination with increased temperatures and water deficit was shown to contribute to the modification of the phenological stages of vines (Martínez-Lüscher et al. 2016).

We found 78 *climate change* *ecosystem condition* *ecosystem service* links from 15 papers. The most studied climate change variables included in these links were temperature (52%) and precipitation (41%), while the most studied ecosystem conditions were water availability (24%), animals and fungi (21%) and landscape composition (15%). Ecosystem services considered in these links were crop production (62%), decomposition and fixing processes (21%), and pest control (18%) (Fig. 4). Like in the *climate change* *ecosystem condition* links, many of the papers studied more than one climate change variable, while single ecosystem conditions and services were considered in the majority of the papers (supplementary figure S2e, f, g). Notably, the number of *climate change* *ecosystem condition* *ecosystem service* links extracted from our sample is increasing in the last few years (Fig. 3c).

Around 20% of the *climate change* *ecosystem condition* *ecosystem service* links were related to the study of temperature and precipitation on water availability, and the consequent effects on crop production. For example, Ramos and Martínez-Casasnovas (2010) studied how temperature and precipitation distributions associated to climate change affect water availability of rainfed vineyards, thus influencing the vine grape yield. Another 19% of the links studied the effects of changed temperature and precipitation patterns on landscape composition, and the related effects on the decomposition and fixing processes provided by VLs. Muñoz-Rojas et al. (2015) studied how soil organic carbon is influenced by changes in temperature and precipitation, which in turn affects carbon stocks. The influences of temperature and precipitation patterns were studied by 20% of the links in relation to the phenology and climatic suitability of vines to determine how they influence the provision of wine grapes, e.g., in Fraga et al. (2016), Fraga et al. (2019). Finally, 9% of the links focused on the effects of changing temperatures on the animals and fungi present in VLs. For example, as illustrated by Thiéry et al. (2018), the increase of temperature influences the abundance and diversity of natural enemies and parasitoids of vine pests in vineyards, affecting their capacity to provide pest control.

### Disciplines

**Fig. 5:**
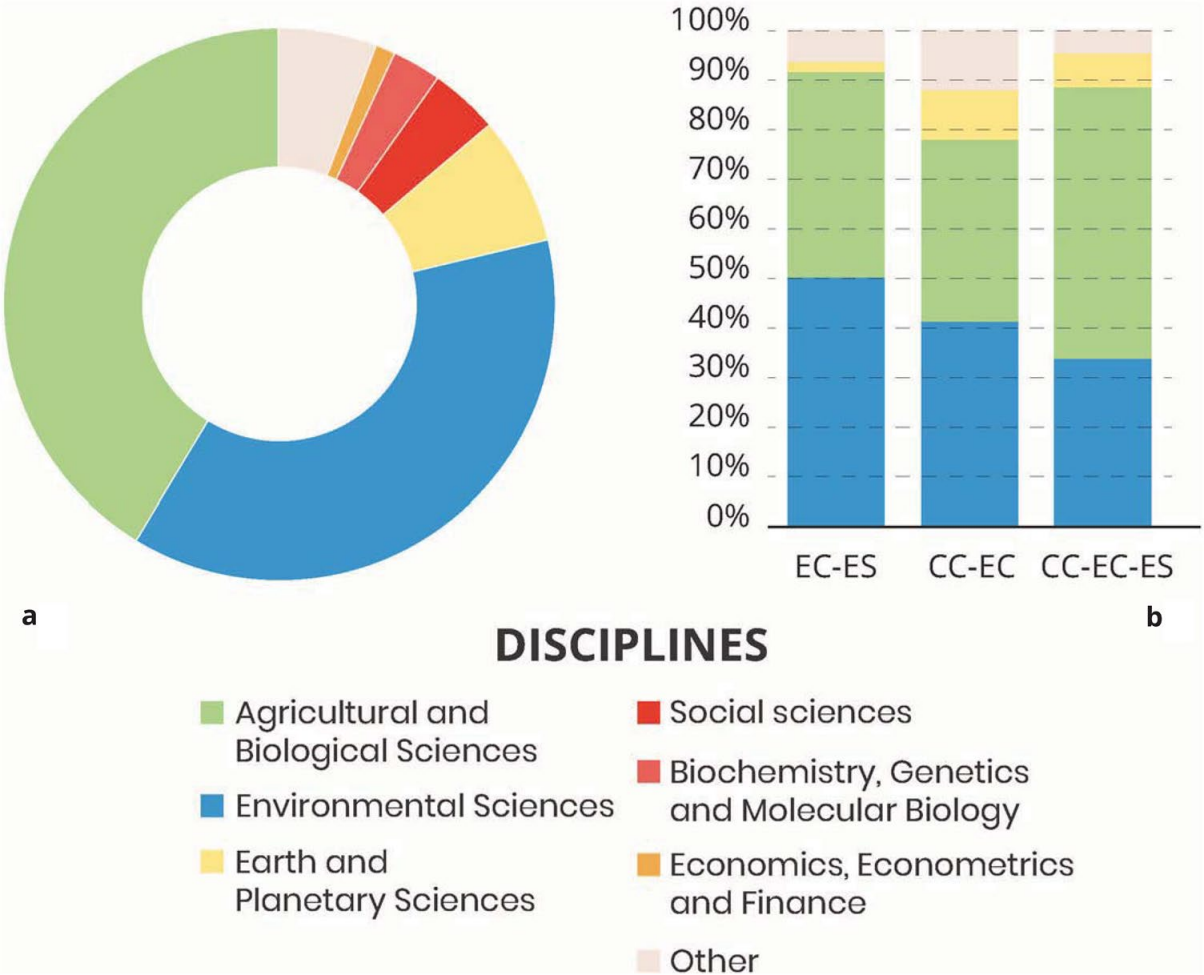
Disciplines included in our review: **a** categorization of the articles in our review based on the disciplines of the journal; **b** categorization of the links retrieved in our review based on the discipline of the journal. In both cases, we accounted for journals ranked in more than one discipline by creating an entry for each discipline

The reviewed papers originated from journals belonging to a limited number of disciplines. Around 41% of the articles were categorized as belonging to the agricultural and biological sciences, 37% to the environmental sciences and 7% to earth and planetary sciences. In particular, papers from the agricultural and biological sciences and from the environmental sciences have been studying VLs over time (supplementary figure S3). Other relevant disciplines were those from the social sciences (4%) and from biochemistry, genetics and molecular biology (3%) (Fig. 5a). *Ecosystem conditions* *ecosystem services* links were addressed in papers published mainly by agricultural and biological sciences, environmental sciences, and earth and planetary sciences journals, with more than 90% of the links originating from either the environmental sciences or the agricultural and biological sciences, showing the importance of these fields. *Climate change  ecosystem conditions* links were studied in papers coming out of environmental sciences, and agricultural and biological sciences in almost 80% of the cases. *Climate change* *ecosystem conditions  ecosystem services* links were studied by environmental sciences, and agricultural and biological sciences in almost 90% of the cases.

## Discussion

### Beyond grape provision

Our study highlighted that even if the number of ecosystem conditions and services investigated in our sample of papers has increased in recent years, there is the need to consider ecosystem conditions and ecosystem services more comprehensively. For example, we found that only 3% of the *ecosystem conditions* *ecosystem services* links retrieved by our review addressed cultural services. In addition, only 28 and 18% of the reviewed studies considered more than two ecosystem conditions or services, respectively. There is, therefore, the need to adopt a more comprehensive view both on the variables that are studied, e.g., which ecosystem conditions and services, and on how multiple variables are investigated together, e.g., multiple ecosystem conditions and services.

The results of our review suggest that there is a need to take into better consideration a wider range of ecosystem conditions and services in VLs. To foster more comprehensive research, future studies on VLs should focus on understudied ecosystem conditions such as vine variety, water availability, and the characteristics of vineyard soil, and understudied ecosystem services such as the provision of fibers and materials, water regulation, and erosion control. More attention should moreover be placed on the intangible services provided by VLs and on the potential of VLs for supporting outdoor recreation, and bequest and existence values. For example, there is evidence that assessments of relational values can support the development of policies that leverage farmers’ sense of identity, such as when designing payments for ecosystem services schemes to support biodiversity conservation and soil conservation practices (Allen et al. 2018; Chan et al. 2016). As also found by previous reviews on VLs (Winkler et al. 2017; Paiola et al. 2020), and on other ecosystem types, i.e., forest, coast, arctic or mountain (Mengist and Soromessa 2019; Liquete et al. 2013; Falardeau and Bennett 2019; Mengist et al. 2019), literature on ecosystem services often focuses on the restricted range of services that are involved in the production of material goods and can be easily quantified (Martín-Lopez et al. 2012). Thus, approaches that study agroecosystems without an exclusively instrumental viewpoint, but that also include intrinsic and relational values, should be adopted more frequently (Himes and Muraca 2018). A more comprehensive approach to the study of VLs could, therefore, provide critical information that can foster the transformation and adaptation of these socio-ecological system.

Our review has moreover found that the majority of articles that study ecosystem services in VLs investigate a single ecosystem condition or ecosystem service. In some cases, there are scientific and practical reasons why a study would focus on a single ecosystem condition. For example, those studies that are aiming to gain an understanding of the functioning of vines under warmer temperatures often look at their phenology because this condition allows the quantification of the differences in the development of the plants starting from the budbreak to the harvest of grape clusters (Fraga et al. 2016, 2019). In other cases, however, focusing on a higher number of ecosystem conditions would allow the better understanding of the interplay of ecosystem services at the ecosystem and landscape scale. In our review, we found only six papers that studied more than two ecosystem conditions and services together. This is the case of Capó-Bauçà et al. (2019) who analyzed three ecosystem services (decomposition and fixing processes by soil, water regulation, the provision of populations and habitats) and two ecosystem conditions (ground cover conditions, vineyard soil) to highlight the benefits provided by green cover crops in Mediterranean vineyards. The other five papers were literature reviews, and this is the reason why a high number of ecosystem conditions and services was included in their study. These reviews were moreover investigating widely studied ecosystem services and conditions, such as ground cover conditions, the presence of animal and fungi, the provision of population and habitats, and decomposition and fixing processes by soil. More comprehensive research on VLs could be fostered by including those ecosystem conditions that are very important for providing multiple services. For instance, in our review local habitat conditions were studied only in a limited number of papers, despite the fact that they affect a wide range of ecosystem services such as pest control, soil quality, and decomposition and fixing processes (Polyakov et al. 2019; Rosas-Ramos et al. 2019; Tixier et al. 2015). Moreover, applying a holistic perspective to address climate change challenges, such as changes in precipitation patterns, could foster the simultaneous study of all the ecosystem conditions and services involved in the process, e.g., in the water cycle. Given the lack of multifunctional research in VLs, conducting research that sheds light on the relationships among the understudied components of VLs would be a first step towards a more complete understanding of these socio-ecological systems (Rusch et al. 2022).

While the study of VLs’ multifunctionality helps to grasp the agroecosystems’ capacity to support various aspects of ecosystem resilience and of human well-being, attention should be placed on the issues related to its assessment methods. Indeed, since only a subset of all the possible functions and services present in an ecosystem can be quantified, multifunctionality measures are not absolute and depend on the ecosystem services or functions included in the studies (Manning et al. 2018). Multifunctionality can be described by different indices which underline the total supply (e.g., average) or the diversity (e.g., alpha diversity) of multiple ecosystem services at different scales (Manning et al. 2018). In addition, some approaches might be better suited than others to consider the trade-offs and synergies occurring between ecosystem services (Schaafsma and Bartkowski 2020). To overcome these gaps, the concept of multifunctionality should be approached carefully, adopting specific methodological steps that can limit some of its drawbacks e.g., incorporating the most important ecosystem services and weighting them based on stakeholders’ priorities (Neyret et al. 2021). In many cases, as suggested by Giling et al. (2018), the methods to assess ecosystem multifunctionality may need to be selected on a case‐by‐case basis developing tailored hypotheses, functions, and analytical methods (Giling et al. 2018). To conclude, the considerations of multiple ecosystem conditions and ecosystem services would enable a systematic deepening of our knowledge on the functions and services provided by VLs, advancing our capacity to manage them.

### Promoting multidisciplinary research in viticulture

Our review highlighted the diverse disciplinary and methodological perspectives adopted in studying ecosystem conditions, ecosystem services, and the effects of climate change on VLs. Although VLs were also investigated by studies published in journals classified in the fields of economics, earth and planetary sciences, social sciences, and other fields, 78% of the reviewed papers have been published by the agricultural and biological sciences and by the environmental sciences for almost 20 years. For example, we found many papers that used a crop modeling approach to study the effects of climate change on yield (such as Fraga et al. 2019), or studies that used a landscape ecology lens to investigate the role of landscape composition in influencing pest control in VLs (such as Rusch et al. 2017). On the other hand, papers included in our sample and that were classified in the discipline category of multidisciplinary studies were rare and included only a few links.

The lack of multidisciplinary approaches in the study of the provision of ecosystem services has been reported in previous reviews about agricultural and other ecosystems (Tancoigne et al. 2014; Liquete et al. 2013; Vári et al. 2022). Indeed, applying multidisciplinarity is often difficult for several reasons: limited funding allocation due to a traditional academic structure that discourages multidisciplinary collaboration, practical challenges related to the management of resources and researchers, and methodological challenges related to the connection between disciplines or the use of established ways to approach a research theme (Pooley et al. 2014; Thompson et al. 2020; Dick et al. 2016). However, having a multidisciplinary approach is important considering the challenges that VLs will face due to climate change and the fact that these challenges are characterized by a high degree of complexity due to the interactions of climate change with other drivers of change (Lopez-Bustins et al. 2013; de Herralde et al. 2010).

To overcome the lack of multidisciplinarity in literature on VLs, communication between different disciplines should be fostered more. A first step towards this target would be the creation of a common vocabulary and shared definitions of the most important concepts that are relevant for VLs. In our review, we found that the concept of ecosystem services has not yet penetrated the journals of agronomy and biological sciences that deal with viticulture, and that some papers had no reference to this concept at all. For example, Fraga et al. (2016) and Gristina et al. (2019) studied crop production and carbon sequestration without explicitly considering them as ecosystem services. Where the ecosystem services concept is used explicitly, several different definitions and classifications are adopted, if specified at all. The same holds for ecosystem conditions, for which we had to rely on a general classification developed for studying agroecosystems at the European scale that we had to adapt to the specific case of viticulture (Maes et al. 2018). In fact, also in papers that included many ecosystem conditions, e.g., Thiéry et al. 2018, we did not find any reference to a common definition or classification system. Future research should, therefore, lay down a shared definition of ecosystem conditions and services for VLs to facilitate a common understanding on these concepts and to advance our understanding of viticulture in a multidisciplinary perspective.

The combination of mixed methods in single studies is another approach that can foster the production of new multidisciplinary knowledge. This is facilitated when research is conducted by a heterogeneous team with different backgrounds (O’Cathain et al. 2008). In our review, we found nine studies that applied mixed methods coupling field observations with experiments or models, and three studies that coupled questionnaires with models or field observations. While the papers that coupled field observation with experiments or models were mainly doing this inside the boundaries of the same discipline, for example comparing results from a field survey with a controlled experiment in the same vineyard plot, the combination of models and questionnaires was done applying a ‘true’ multidisciplinary perspective. Some of the studies that coupled models and questionnaires shed light on specific aspects of VLs that would have been hardly grasped using a monodisciplinary focus. For example, Lereboullet et al. 2014 complemented present climate data and its projections with in-depth interviews of local stakeholders to analyze the adaptive capacity of the Languedoc–Roussillon winemaking region (FR). Holland and Smit 2014 applied a similar approach to evaluate the adaptation strategies that are employed by wine producers in Prince Edward County (CA). These methods can increase the knowledge of VLs as a socio-ecological system and can foster the adoption of strategies that support climate change adaptation. In addition, such multidisciplinary methods could be applied also to study other drivers that are increasingly threatening VLs, e.g., socio-economic pressures, land use change, and biodiversity loss (Viers et al. 2013; Hoppert et al. 2018). To conclude, adopting methods that are used in different disciplines, such as questionnaires and models, should be prioritized in the future, as they can increase the multidisciplinary knowledge on VLs.

### Benefits of an integrative perspective

Our findings highlighted that integrative studies, i.e., studies that included *climate change  ecosystem conditions  ecosystem services* links, were underrepresented in the literature. Out of the total 476 links retrieved in our review, we found that only 78 were integrative. Of the fifteen studies that included integrative links, seven were literature reviews (including more than 50% of all the integrative links), and six were research papers. The ecosystem services addressed by such integrative studies were provisioning in 62% of the cases and regulating in 38%. Of the fifteen ecosystem conditions considered in this review, only eight were addressed in these types of studies.

To foster studies with an integrative perspective it would be important to better investigate some specific ecosystem conditions that have the potential to develop integrative knowledge. This is the case of ecosystem conditions such as the presence of animals and fungi, and vineyard soil that were considered in both *climate change* *ecosystem conditions* and *ecosystem conditions* *ecosystem services* links but were not addressed by integrative studies. Indeed, since knowledge about these two separate link types has been already produced for these ecosystem conditions, this knowledge can be the basis for developing an integrative understanding of the relationship between climate change attributes and ecosystem services. Another way to develop more integrative knowledge would be to study those ecosystem conditions and services that have not yet been assessed in studies featuring *climate change* *ecosystem conditions* *ecosystem services* links. For instance, ground cover conditions and local scale habitat conditions have never been studied in relation to both climate change attributes and ecosystem services, and we did not find any integrative link that included cultural ecosystem services. The inclusion of these conditions and services in integrative studies would complement the many studies already available on *ecosystem conditions* *ecosystem services* links, underlying the role of climate change in regulating such biophysical processes and ecosystem services provision.

Integrative research papers can provide critical information on the cascading effects that would be difficult to grasp focusing only on parts of the socio-ecological system, i.e., the other two link types (Falardeau and Bennett 2019). This knowledge allows researchers and decision makers to shed light on the functioning of each VL, supporting the development of strategies that can shape more resilient and sustainable VLs. This is particularly important in VLs that are often distinguished based on the concept of terroir, which defines the unique aspects of each growing region (Winkler et al. 2017). For example, those *climate change* *ecosystem condition* *ecosystem service* links that studied the effects of climate change on the climatic suitability of vines and the related effects on yield in specific winegrowing areas may constitute an entry point for the identification of possible adaptation options that take into consideration the complexity of each VL. This is the case of the study of Biasi et al. (2019), which characterized the genotypic‐specific response to climate change of a set of local and international vine varieties in the Umbria Region (IT) and thus enabled the development of viticultural practices in line with the local climate. Future studies on the specific consequences of climate change on ecosystem conditions and services should, therefore, be promoted to foster the development of tailored regional adaptation strategies.

### Further developments

Although we mapped how extensively the relations between the VL components were studied, our approach did not quantify the impacts of climate change on the ecosystem conditions and services. Future studies could conduct a dedicated meta-analysis based on the data included in the literature. Future studies can also expand our approach including additional information on the relationships between ecosystem conditions, ecosystem services, and climate change in VLs by including possible feedbacks occurring between these components, as we considered only the description of direct effects of ecosystem conditions on ecosystem services, and of a set of climate change attributes on them. For example, we did not study the effects of ecosystem conditions on other ecosystem conditions, although, for instance, certain management practices may impact other sets of conditions by using fewer external inputs (e.g., in organic farming). These influences of the ecosystem conditions can boost the sustainability of a VL and increase the presence of beneficial organisms such as natural enemies of crop pests (Muneret et al. 2019). Even though we excluded human-interacting feedbacks in VLs, some of the reviewed papers considered specific socio-economic drivers together with climate change in the analysis of VLs (Bernardo et al. 2018; Castex et al. 2015; Neethling et al. 2017; Sgubin et al. 2019). In these papers, however, it was difficult to identify how such socio-economic drivers would affect the provision of ecosystem services in VLs. The use of system-thinking methods and tools such as causal loop diagrams and stock-flow models, which have been successfully applied to study complex problems related to agroecosystems, could be effective for analyzing the complexity of VLs including human dimensions (Sterman 2000; Turner et al. 2016; Walters et al. 2016). The application of these methods could promote a holistic understanding of agricultural landscapes, their environment, and food production, which will be essential to meet policy objectives such as the sustainable development goals (Ortiz et al. 2021).

## Conclusions

VLs are important agroecosystems that provide multiple economic, environmental, and cultural ecosystem services. The literature on VLs, however, is missing a comprehensive, multidisciplinary, and integrative approach to the study of ecosystem services and conditions in the context of climate change. To fill this gap and to promote more multifunctional approaches in the study of VLs, future research should focus on the least studied ecosystem conditions (e.g., vineyard soil characteristics, vine variety) and services (e.g., cultural ecosystem services), with a focus on those key ecosystem conditions that are linked to the provision of multiple ecosystem services. In addition, more efforts should be put in developing common definitions for key ecosystem services and conditions in viticulture and in applying mixed methods to study VLs. This would foster the production of new knowledge that crosses the boundaries of single disciplines. Finally, to develop more integrative research on VLs, attention should be placed on the ecosystem conditions that can potentially link existing knowledge on climate change and on ecosystem services. The knowledge developed in such comprehensive, multidisciplinary, and integrative studies will help researchers and decision makers to gain a more complete understanding of agroecosystems’ overall functioning and to identify effective adaptation strategies that can support the sustainable management of VLs under future climate uncertainties.

## Supplementary Information

Below is the link to the electronic supplementary material.

Supplementary file1 (DOCX 921 KB)
