# Supplementary material for: An ecosystem service approach to the study of vineyard landscapes in the context of climate change: a review
Source: Sustain Sci. 2022 Sep 17;18(2):997–1013. doi: 10.1007/s11625-022-01223-x (PMC10063506; doi:10.1007/s11625-022-01223-x)
Supplement: Supplementary file 1 — Supplementary file1 (DOCX 921 KB) [file 11625_2022_1223_MOESM1_ESM.docx]

**An ecosystem service approach to the study of vineyard landscapes in the context of climate change: a review**

Sebastian Candiago ^a,b^, Klara Johanna Winkler ^c^, Valentina Giombini ^a^, Carlo Giupponi ^b^, Lukas Egarter Vigl ^a^

^a^ Institute for Alpine Environment, Eurac Research, Viale Druso 1, 39100 Bozen/Bolzano, Italy

^b^ Ca' Foscari University of Venice, Department of Economics, S. Giobbe 873, 30121 Venezia, Italy

^c^ McGill University, Macdonald Campus, 21,111 Lakeshore Drive, Ste-Anne-de-Bellevue, Québec, H9X 3V9, Canada

Corresponding author: [sebastian.candiago@eurac.edu](mailto:sebastian.candiago@eurac.edu)

**Abstract**

Vineyard landscapes significantly contribute to the economy, identity, culture, and biodiversity of many regions worldwide. Climate change, however, is increasingly threatening the resilience of vineyard landscapes and of their ecological conditions, undermining the provision of multiple ecosystem services. Previous research has often focused on climate change impacts, ecosystem conditions and ecosystem services without systematically reviewing how they have been studied in the literature on viticulture. Here, we systematically review the literature on vineyard landscapes to identify how ecosystem conditions and services have been investigated, and whether an integrative approach to investigate the effects of climate change was adopted. Our results indicate that there are still very few studies that explicitly address multiple ecosystem conditions and services together. Only 28% and 18% of the reviewed studies considered more than two ecosystem conditions or services, respectively. Moreover, while more than 97% of the relationships between ecosystem conditions and services studied were addressing provisioning and regulating services, only 3% examined cultural services. Finally, this review found that there is a lack of integrative studies that address simultaneously the relationships between ecosystem condition, ecosystem services and climate change (only 15 out of 112 studies). To overcome these gaps and to better understand the functioning of vineyard socio-ecological systems under climate change, multidisciplinary, integrative, and comprehensive approaches should be adopted by future studies. A holistic understanding of vineyard landscapes will indeed be crucial to support researchers and decision makers in developing sustainable adaptation strategies that enhance the ecological condition of vineyards and ensure the provision of multiple ecosystem services under future climate scenarios.

**Keywords**

Viticulture, Agricultural System, Socio-ecological system, Ecological Condition, Global Warming, Adaptation

## **Tables**

**Table S1:** strings used for the search papers with a focus on: (i) climate change effects on ecosystem conditions in vineyard landscapes and (ii) the ecosystem services provided by vineyard landscapes.

| **Focus** | **Search strings** |
| --- | --- |
| **(i) Climate change effects on ecosystem conditions in vineyard landscapes** (keywords are based on the indicators suggested for agroecosystems in Maes et al. (2018) | ((vineyard OR viticulture OR "grape* grow*" OR wine*) AND ("climat* change*" OR "global change" OR "environmental change" OR "climat* warm*" OR "global warm*" OR "temperature rise" OR "extreme event*" OR "extreme weather" OR "greenhouse gas*" OR "global emissions") AND (*nitrogen OR "heavy metal" OR fragmentation OR diversity OR rotation OR density OR connectivity OR "semi-natural" OR seminatural OR fallow OR "high nature value*" OR organic OR livestock* OR bird* OR mammal* OR amphibian* OR reptile* OR pollinator* OR habitat OR "protected area*" OR "soil organic carbon" OR "soil pH" OR "soil erodibility" OR "bulk density" OR "soil biodiversity" OR "water availability" OR "gross primary production" OR "water capacity" OR "soil nutrient*"*)) |
| **(ii) Ecosystem services in vineyard landscapes** | ((vineyard OR viticulture OR "grape* grow*" OR wine*) AND (*"ecosystem service*"*)) |

**Table S2:** strings used for the search of the articles regarding climate change effects on ecosystem services provided by vineyard landscapes and the influence of ecosystem conditions. In this study we considered ecosystem services classes from the CICES classification v5.1 (adapted from Haines-Young and Potschin (2018), CC BY 4.0). For the selection of the 28 CICES classes to be studied for vineyard landscapes and the related keywords used in the search we referred to (Winkler et al. 2017).

| **Ecosystem service searched** | **CICES 5.1 code** | **Search string** |
| --- | --- | --- |
| Cultivated terrestrial plants (including fungi, algae) grown for nutritional purposes | 1.1.1.1 | ((vineyard OR viticulture OR "grape* grow*" OR wine*) AND ("climat* change*" OR "global change" OR "environmental change" OR "climat* warm*" OR "global warm*" OR "temperature rise" OR "extreme event*" OR "extreme weather" OR "greenhouse gas*" OR "global emissions") AND (winemaker* OR winegrower* OR farmer* OR producer* OR vintner*) AND (*yield* OR "grape leaves" OR "grapevine leaves" OR crop* OR "table grape*" OR "crop load*" OR "grape berr*" OR "berry growth" OR "grape maturity" OR "yield component*" OR "fruit composition" OR "cultivated crops*" OR "wine grape*" OR "grape juice" OR wine OR "sugar content"*)) |
| Fibres and other materials from cultivated plants, fungi, algae and bacteria for direct use or processing (excluding genetic materials) | 1.1.1.2 | ((vineyard OR viticulture OR "grape* grow*" OR wine*) AND ("climat* change*" OR "global change" OR "environmental change" OR "climat* warm*" OR "global warm*" OR "temperature rise" OR "extreme event*" OR "extreme weather" OR "greenhouse gas*" OR "global emissions") AND (winemaker* OR winegrower* OR farmer* OR producer* OR vintner*) AND (*pruning OR "grape seed*" OR "grape skin*" OR MegaPurple OR "color additive*" OR wood OR "Ravaz index"*)) |
| Fibres and other materials from reared animals for direct use or processing (excluding genetic materials) | 1.1.3.2 | ((vineyard OR viticulture OR "grape* grow*" OR wine*) AND ("climat* change*" OR "global change" OR "environmental change" OR "climat* warm*" OR "global warm*" OR "temperature rise" OR "extreme event*" OR "extreme weather" OR "greenhouse gas*" OR "global emissions") AND (winemaker* OR winegrower* OR farmer* OR producer* OR vintner*) AND (*pomace*)) |
| Filtration/sequestration/storage/accumulation by micro-organisms, algae, plants, and animals | 2.1.1.2 | ((vineyard OR viticulture OR "grape* grow*" OR wine*) AND ("climat* change*" OR "global change" OR "environmental change" OR "climat* warm*" OR "global warm*" OR "temperature rise" OR "extreme event*" OR "extreme weather" OR "greenhouse gas*" OR "global emissions") AND (winemaker* OR winegrower* OR farmer* OR producer* OR vintner*) AND *("carbon storage" OR "carbon sequestration" OR filtration OR sequestration OR storage OR accumulation OR GHG OR "greenhouse gas" OR N2O OR "nitrous oxide" OR sulfur OR "nitrogen deposition*" OR fertilizer* OR spray OR pesticide* OR salinization OR "soil salinity" OR "salt accumulation"*)) |
| Smell reduction | 2.1.2.1 | ((vineyard OR viticulture OR "grape* grow*" OR wine*) AND ("climat* change*" OR "global change" OR "environmental change" OR "climat* warm*" OR "global warm*" OR "temperature rise" OR "extreme event*" OR "extreme weather" OR "greenhouse gas*" OR "global emissions") AND (winemaker* OR winegrower* OR farmer* OR producer* OR vintner*) AND *("spatial planning" OR "land use planning" OR "smell impact" OR smell OR "sulfur smell" OR harvest OR "crush smell"*)) |
| Noise attenuation | 2.1.2.2 | ((vineyard OR viticulture OR "grape* grow*" OR wine*) AND ("climat* change*" OR "global change" OR "environmental change" OR "climat* warm*" OR "global warm*" OR "temperature rise" OR "extreme event*" OR "extreme weather" OR "greenhouse gas*" OR "global emissions") AND (winemaker* OR winegrower* OR farmer* OR producer* OR vintner*) AND (*"spatial planning" OR "land use planning" OR "noise impact" OR "tractor noise" OR "sound cannon*" OR highway*)) |
| Visual screening | 2.1.2.3 | ((vineyard OR viticulture OR "grape* grow*" OR wine*) AND ("climat* change*" OR "global change" OR "environmental change" OR "climat* warm*" OR "global warm*" OR "temperature rise" OR "extreme event*" OR "extreme weather" OR "greenhouse gas*" OR "global emissions") AND (winemaker* OR winegrower* OR farmer* OR producer* OR vintner*) AND (*"spatial planning" OR "land use planning" OR zoning OR reflector OR "visual impact" OR landscape OR viewshed OR preservation OR aesthetics*)) |
| Control of erosion rates | 2.2.1.1 | ((vineyard OR viticulture OR "grape* grow*" OR wine*) AND ("climat* change*" OR "global change" OR "environmental change" OR "climat* warm*" OR "global warm*" OR "temperature rise" OR "extreme event*" OR "extreme weather" OR "greenhouse gas*" OR "global emissions") AND (winemaker* OR winegrower* OR farmer* OR producer* OR vintner*) AND *("soil conservation" OR "soil loss*" OR "cultivation practice*" OR "mass stabilization" OR erosion OR "erosion rate" OR "erosion model" OR "alternate row cultivation" OR "row cultivation" OR disking OR mowing OR ripping OR liming OR "tree removal" OR "run off" OR erosivity OR "land terrac*" OR "native vegetation removal" OR "vegetation removal" OR "cover crop" OR "mass flow" OR tractor* OR machinery OR steep slope*)) |
| Hydrological cycle and water flow regulation (Including flood control, and coastal protection) | 2.2.1.3 | ((vineyard OR viticulture OR "grape* grow*" OR wine*) AND ("climat* change*" OR "global change" OR "environmental change" OR "climat* warm*" OR "global warm*" OR "temperature rise" OR "extreme event*" OR "extreme weather" OR "greenhouse gas*" OR "global emissions") AND (winemaker* OR winegrower* OR farmer* OR producer* OR vintner*) AND *("fraction of transpirable soil water" OR FTSW OR infiltration OR "water deficit" OR "water relations" OR hydraulics OR "run off" OR soil moisture OR irrigation OR (fish AND flows) OR "ecolog* flow*" OR "water security" OR "water stress" OR flooding OR landscape OR "buffer zone" OR setback OR "flood control" OR "flood protection" OR "wet feet" OR drainage*)) |
| Pollination (or 'gamete' dispersal in a marine context) | 2.2.2.1 | ((vineyard OR viticulture OR "grape* grow*" OR wine*) AND ("climat* change*" OR "global change" OR "environmental change" OR "climat* warm*" OR "global warm*" OR "temperature rise" OR "extreme event*" OR "extreme weather" OR "greenhouse gas*" OR "global emissions") AND (winemaker* OR winegrower* OR farmer* OR producer* OR vintner*) AND (*insect* OR pollination OR bee OR finch* OR "cover crop" OR "wind pollination" OR arthropod*)) |
| Seed dispersal | 2.2.2.2 | ((vineyard OR viticulture OR "grape* grow*" OR wine*) AND ("climat* change*" OR "global change" OR "environmental change" OR "climat* warm*" OR "global warm*" OR "temperature rise" OR "extreme event*" OR "extreme weather" OR "greenhouse gas*" OR "global emissions") AND (winemaker* OR winegrower* OR farmer* OR producer* OR vintner*) AND *("seed dispersal" OR bird* OR starling* OR turkey* OR "sound cannons*"*)) |
| Maintaining nursery populations and habitats (Including gene pool protection) | 2.2.2.3 | ((vineyard OR viticulture OR "grape* grow*" OR wine*) AND ("climat* change*" OR "global change" OR "environmental change" OR "climat* warm*" OR "global warm*" OR "temperature rise" OR "extreme event*" OR "extreme weather" OR "greenhouse gas*" OR "global emissions") AND (winemaker* OR winegrower* OR farmer* OR producer* OR vintner*) AND (*diversity OR biodiversity OR "nursery population" OR habitat OR germplasm OR "biological resource" OR "gene pool"*)) |
| Pest control (including invasive species) | 2.2.3.1 | ((vineyard OR viticulture OR "grape* grow*" OR wine*) AND ("climat* change*" OR "global change" OR "environmental change" OR "climat* warm*" OR "global warm*" OR "temperature rise" OR "extreme event*" OR "extreme weather" OR "greenhouse gas*" OR "global emissions") AND (winemaker* OR winegrower* OR farmer* OR producer* OR vintner*) AND *("cover crop" OR pest* OR "pest control*" OR "rodent control*" OR "beneficial predator*" OR "bird box*" OR "owl box*" OR "raptor box*" OR "nest box*" OR "integrated pest management*" OR IPM OR "native plant*" OR "natural enemy" OR "pest management" OR pesticide OR "biological control" OR arthropod OR rodent* OR insecticide* OR phylloxera OR nematode**)) |
| Disease control | 2.2.3.2 | ((vineyard OR viticulture OR "grape* grow*" OR wine*) AND ("climat* change*" OR "global change" OR "environmental change" OR "climat* warm*" OR "global warm*" OR "temperature rise" OR "extreme event*" OR "extreme weather" OR "greenhouse gas*" OR "global emissions") AND (winemaker* OR winegrower* OR farmer* OR producer* OR vintner*) AND *("red blotch" OR botrytis OR fungal OR herbicide OR phomopsis OR disease* OR fungicide*OR disorder*OR eutypa OR "biological control" OR fanleaf OR mulch OR leafroll OR "corky bark"*)) |
| Weathering processes and their effect on soil quality | 2.2.4.1 | ((vineyard OR viticulture OR "grape* grow*" OR wine*) AND ("climat* change*" OR "global change" OR "environmental change" OR "climat* warm*" OR "global warm*" OR "temperature rise" OR "extreme event*" OR "extreme weather" OR "greenhouse gas*" OR "global emissions") AND (winemaker* OR winegrower* OR farmer* OR producer* OR vintner*) AND *("soil fertility" OR nutrient* OR "soil structure" OR "in situ soil" OR "soil biological activity" OR "nutrient uptake" OR mineral* OR "soil quality" OR "weathering process*"*)) |
| Decomposition and fixing processes and their effect on soil quality | 2.2.4.2 | ((vineyard OR viticulture OR "grape* grow*" OR wine*) AND ("climat* change*" OR "global change" OR "environmental change" OR "climat* warm*" OR "global warm*" OR "temperature rise" OR "extreme event*" OR "extreme weather" OR "greenhouse gas*" OR "global emissions") AND (winemaker* OR winegrower* OR farmer* OR producer* OR vintner*) AND (*microbe* OR fungi OR "soil arthropod*" OR arthropod OR mulch OR worm* OR legume* OR "nitrogen fixing" OR "soil quality" OR decomposition OR "fixing process*"*)) |
| Regulation of temperature and humidity, including ventilation and transpiration | 2.2.6.2 | ((vineyard OR viticulture OR "grape* grow*" OR wine*) AND ("climat* change*" OR "global change" OR "environmental change" OR "climat* warm*" OR "global warm*" OR "temperature rise" OR "extreme event*" OR "extreme weather" OR "greenhouse gas*" OR "global emissions") AND (winemaker* OR winegrower* OR farmer* OR producer* OR vintner*) AND *("latent heat" OR transpiration OR "climat* regulation" OR shade OR "hydrologic cycle" OR "micro climate" OR "regional climate" OR evapotranspiration OR ventilation OR photosynthesis OR ecophysiology*)) |
| Characteristics of living systems that enable activities promoting health, recuperation or enjoyment through active or immersive interactions | 3.1.1.1 | ((vineyard OR viticulture OR "grape* grow*" OR wine*) AND ("climat* change*" OR "global change" OR "environmental change" OR "climat* warm*" OR "global warm*" OR "temperature rise" OR "extreme event*" OR "extreme weather" OR "greenhouse gas*" OR "global emissions") AND (winemaker* OR winegrower* OR farmer* OR producer* OR vintner*) AND *("wine tasting" OR picnic* OR "eating grape*" OR "drink* wine" OR dolmade* OR birding OR "bird watch*" OR employment OR "hot air" OR "balloon ride" OR "limousin* tour*" OR "gourmet tourism" OR "cable car"*)) |
| Characteristics of living systems that enable activities promoting health, recuperation or enjoyment through passive or observational interactions | 3.1.1.2 | ((vineyard OR viticulture OR "grape* grow*" OR wine*) AND ("climat* change*" OR "global change" OR "environmental change" OR "climat* warm*" OR "global warm*" OR "temperature rise" OR "extreme event*" OR "extreme weather" OR "greenhouse gas*" OR "global emissions") AND (winemaker* OR winegrower* OR farmer* OR producer* OR vintner*) AND (*biking OR hiking OR "horseback rid*" OR padding OR walking*)) |
| Characteristics of living systems that enable scientific investigation or the creation of traditional ecological knowledge | 3.1.2.1 | ((vineyard OR viticulture OR "grape* grow*" OR wine*) AND ("climat* change*" OR "global change" OR "environmental change" OR "climat* warm*" OR "global warm*" OR "temperature rise" OR "extreme event*" OR "extreme weather" OR "greenhouse gas*" OR "global emissions") AND (winemaker* OR winegrower* OR farmer* OR producer* OR vintner*) AND *("climate change" OR enology OR trial OR "precision viticulture" OR scientific*)) |
| Characteristics of living systems that enable education and training | 3.1.2.2 | ((vineyard OR viticulture OR "grape* grow*" OR wine*) AND ("climat* change*" OR "global change" OR "environmental change" OR "climat* warm*" OR "global warm*" OR "temperature rise" OR "extreme event*" OR "extreme weather" OR "greenhouse gas*" OR "global emissions") AND (winemaker* OR winegrower* OR farmer* OR producer* OR vintner*) AND (*winemaking OR winegrowing OR "wine seminar" OR school OR university OR college OR education OR "tasting room" OR "environmental education"*)) |
| Characteristics of living systems that are resonant in terms of culture or heritage | 3.1.2.3 | ((vineyard OR viticulture OR "grape* grow*" OR wine*) AND ("climat* change*" OR "global change" OR "environmental change" OR "climat* warm*" OR "global warm*" OR "temperature rise" OR "extreme event*" OR "extreme weather" OR "greenhouse gas*" OR "global emissions") AND (winemaker* OR winegrower* OR farmer* OR producer* OR vintner*) AND *("family winery" OR tradition OR charm OR traditional OR historical OR identity OR "sense of place" OR "social capital" OR heritage OR "local food cultural"*)) |
| Characteristics of living systems that enable aesthetic experiences | 3.1.2.4 | ((vineyard OR viticulture OR "grape* grow*" OR wine*) AND ("climat* change*" OR "global change" OR "environmental change" OR "climat* warm*" OR "global warm*" OR "temperature rise" OR "extreme event*" OR "extreme weather" OR "greenhouse gas*" OR "global emissions") AND (winemaker* OR winegrower* OR farmer* OR producer* OR vintner*) AND (*beauty OR scenery OR landscape OR winescape OR "vineyard row" OR aesthetic OR mustard OR poppies OR inspiration OR wildflower OR "seasonal change" OR "leaf change" OR "foliage change" OR art OR gallery*)) |
| Elements of living systems that have symbolic meaning | 3.2.1.1 | ((vineyard OR viticulture OR "grape* grow*" OR wine*) AND ("climat* change*" OR "global change" OR "environmental change" OR "climat* warm*" OR "global warm*" OR "temperature rise" OR "extreme event*" OR "extreme weather" OR "greenhouse gas*" OR "global emissions") AND (winemaker* OR winegrower* OR farmer* OR producer* OR vintner*) AND (*representation OR appellation OR symbolic OR "social cohesion" OR terroir OR uniqueness OR AVA OR "American Viticultural Area" OR DOC OR "denomination origine controlle" OR "denominazione di origine controllata" OR AOC OR "Appellation d’origine contrôlée" OR emblem**)) |
| Elements of living systems that have sacred or religious meaning | 3.2.1.2 | ((vineyard OR viticulture OR "grape* grow*" OR wine*) AND ("climat* change*" OR "global change" OR "environmental change" OR "climat* warm*" OR "global warm*" OR "temperature rise" OR "extreme event*" OR "extreme weather" OR "greenhouse gas*" OR "global emissions") AND (winemaker* OR winegrower* OR farmer* OR producer* OR vintner*) AND (*wedding* OR yoga OR meditation OR retreat OR spiritual OR sacred OR religious OR religion OR "mother earth" OR inspiration*)) |
| Elements of living systems used for entertainment or representation | 3.2.1.3 | ((vineyard OR viticulture OR "grape* grow*" OR wine*) AND ("climat* change*" OR "global change" OR "environmental change" OR "climat* warm*" OR "global warm*" OR "temperature rise" OR "extreme event*" OR "extreme weather" OR "greenhouse gas*" OR "global emissions") AND (winemaker* OR winegrower* OR farmer* OR producer* OR vintner*) AND (*wedding* OR entertainment OR "bachel* part*" OR "winery tour" OR "wine tasting" OR concert OR theater OR music OR movie* OR "film festival" OR festival OR "harvest festival" OR contest OR "vintage festival" OR "wine queen" OR "wine event*" OR tourism OR agritourism OR agrotourism OR "wine cave" OR "wine tourism" OR "wine tour*" OR visit OR "day trip"*)) |
| Characteristics or features of living systems that have an existence value | 3.2.2.1 | ((vineyard OR viticulture OR "grape* grow*" OR wine*) AND ("climat* change*" OR "global change" OR "environmental change" OR "climat* warm*" OR "global warm*" OR "temperature rise" OR "extreme event*" OR "extreme weather" OR "greenhouse gas*" OR "global emissions") AND (winemaker* OR winegrower* OR farmer* OR producer* OR vintner*) AND (*view OR "land use" OR "option value" OR existence OR "nature conservation" OR landscape*)) |
| Characteristics or features of living systems that have an option or bequest value | 3.2.2.2 | ((vineyard OR viticulture OR "grape* grow*" OR wine*) AND ("climat* change*" OR "global change" OR "environmental change" OR "climat* warm*" OR "global warm*" OR "temperature rise" OR "extreme event*" OR "extreme weather" OR "greenhouse gas*" OR "global emissions") AND (winemaker* OR winegrower* OR farmer* OR producer* OR vintner*) AND *("family farming" OR "family winery" OR "inter-generational" OR stewardship OR "land ethic" OR bequest*)) |

**Table S3**: inclusion and exclusion criteria used during the paper selection. The focus of the articles to be excluded were defined by the authors while screening the titles, abstracts, and keywords of the papers in our sample.

| **Inclusion criteria** |
| --- |
| (1) Article published in a peer-reviewed journal  (2) Paper type is research article or review  (3) Article’s language is English  (4) Focus on past, present, or future conditions  (5) Focus on vineyard landscapes  (6) Papers that study, in both a qualitative or a quantitative perspective, with or without the support of specific data, and including significant and non-significant correlations from empirical studies either: the relationships between climate change and ecosystem conditions; or the relationships between ecosystem conditions and ecosystem services; or the relationship between climate change, ecosystem conditions and ecosystem services. |
| **Exclusion criteria** |
| (1) Focus only on theoretical methodological approaches/technologies/frameworks not explicitly including the relationships from inclusion criteria (6)  (2) Focus on sustainability/energy/waste/economic assessments (life cycle assessment, SWOT, GHG emissions, energy requirements and production, water, firm performance, waste management) not explicitly including the relationships from inclusion criteria (6)  (3) Focus on oenology, berry composition, sensorial profile of wines, analysis of vine genotype, characteristics and specific physiological traits, effects of abandonment on vine and vineyard, experiments performed in mesocosms without any results measured in situ, in vitro/laboratory analysis  (4) Focus on policy, conceptual frameworks, vulnerability, adaptive capacity, adaptation strategies(5) Study focus is not on vineyard landscapes, or it is neither on a relevant ecosystem condition nor on a relevant ecosystem service for VL |

**Table S4:** options used to answer the structured questions of our review.

| **Question Id** | **Options for answer** |
| --- | --- |
| Q1 | Type of article:   - **Research paper**   - **Model =** a method based on a numerical model to analyse VLs.   - **Field experiment =** an experiment that include the manipulation of some field conditions.   - **Field observation =** a method based on in situ observations.   - **Questionnaire =** a method that include the compilation of a survey. - **Review**   - **Literature review =** a review of the available literature on a certain field. |
| Q2 | - **Local** = study focuses at big geographical scale including one or a few vineyard plots nearby. - **Regional** = study focuses on a larger number of vineyard plots in the same region, or on vineyard plots in different regions from the same country, or it includes a whole viticultural region. - **National** = study focuses on the different vineyard regions in a country. - **Transnational** = study focuses on very small geographical scale or it includes plots from different countries. - **NA** = used for review papers. |
| Q3 | - **Observed** = study focuses on observed features of the analysed phenomenon. - **Future** = study includes future projection of the analysed phenomenon. - **NA =** used for review papers. |
| Q4 | - **Yes** = the study included only *ecosystem conditions 🡪 ecosystem services* links. - **No** = the study included *climate change 🡪ecosystem conditions*, or a *climate change 🡪 ecosystem conditions 🡪 ecosystem services* links. |
| Q5 | - **Yes** = the study included *climate change 🡪ecosystem conditions*, or a *climate change 🡪 ecosystem conditions 🡪 ecosystem services* links. - **No** = the study included only *ecosystem conditions 🡪 ecosystem services* links. |
| Q6 | The list of disciplines involved in the study of ecosystem conditions and services that were included in this study is provided in **Table S6**. |
| Q7 | The list of disciplines involved in the study of climate change variables that were included in this study is provided in **Table S6**. |
| Q8 | The list of normalized ecosystem conditions considered in this study is provided in **Table S5**. |
| Q9 | The list of ecosystem services considered in this study is provided in **Table S2**. |
| Q10 | The *ecosystem conditions 🡪 ecosystem services* links studied in our sample are presented in **Figure 4**. |
| Q11 | The general climate change effects considered in this study are related to temperature, precipitation, CO_2_ concentration or extreme events (as defined in the study). |
| Q12 | The *climate change 🡪ecosystem conditions*, or *climate change 🡪 ecosystem conditions 🡪 ecosystem services* links are presented in **Figure 4**. |

**Table S5**: ecosystem conditions classes considered in this review. To determine the ecosystem conditions studied in each article, we analysed the paper listing the cropland agroecosystem conditions as defined by the framework developed for the mapping and assessment of ecosystem and their services (MAES) (Maes et al. 2018). Specific ecosystem conditions related to vineyard landscapes found during the screening of the articles, e.g., vine variety and vine pruning technique, were added to the list of ecosystem conditions addressed. We grouped ecosystem conditions into homogeneous classes to reconcile different terminologies used to describe the same ecosystem conditions in different fields and to simplify the integration of knowledge with respect to climate change and ecosystem services.

| **Ecosystem conditions from single papers** | **Normalized classes** |
| --- | --- |
| Degree of bird functional diversity | Animals and fungi |
| Host density |  |
| Presence of pests |  |
| Presence of A. Pseudococci |  |
| Presence of arbuscular mycorrhiza |  |
| Presence of arthropods |  |
| Presence of bats |  |
| Presence of birds |  |
| Presence of C. Montrouzieri |  |
| Presence of carabids |  |
| Presence of H. Dimidiatus |  |
| Presence of diseases |  |
| Presence of dominant invasive predator |  |
| Presence of fungi |  |
| Presence of insects |  |
| Presence of L. Abnormis |  |
| Presence of L. Botrana |  |
| Presence of E. Ambiguella |  |
| Presence of natural enemies |  |
| Presence of spiders |  |
| Suitability for grape growing | Climatic suitability |
| Suitability for viticulture |  |
| Degree of berries growth | Gross primary production |
| Grapevine vigor |  |
| Presence of buds |  |
| Status of buds and trunk development |  |
| Status of vegetative development |  |
| Quantity of biomass |  |
| Presence of bare inter-rows | Ground cover |
| Presence of biochar |  |
| Presence of compost |  |
| Presence of cover crops in inter-row space |  |
| Diversely structured inter-row vegetation |  |
| Diversity of cover crops |  |
| Extensive inter-row management |  |
| Availability of floral resources in inter-rows |  |
| Presence of flower driven cover crop |  |
| Presence of grass cover |  |
| Presence of green cover crop |  |
| Ground cover diversity |  |
| Presence of inter-row grass cover |  |
| Presence of inter-row herbaceous cover |  |
| Presence of mulched P. Tanacetifolia and ryegrass |  |
| Presence of mulch |  |
| Presence of native cover crop |  |
| Presence of native ground vegetation |  |
| Presence of natural green cover |  |
| Presence of amendment |  |
| Presence of compost |  |
| Presence of cover crops |  |
| Presence of extensive inter-row vegetation |  |
| Presence of sediment barriers |  |
| Use of service crops |  |
| Presence of tilled inter-rows |  |
| Presence of spontaneous cover crop |  |
| Presence of heat stress | Heat and water stress |
| Presence of water stress |  |
| Increase of artificial areas | Landscape composition |
| Diversity of agricultural crops |  |
| Land cover diversity |  |
| Land cover heterogeneity |  |
| Land cover type |  |
| Land use change |  |
| Landscape complexity |  |
| Landscape diversity |  |
| Landscape fragmentation |  |
| Land use changed to hay crop |  |
| Land use changed to grassed vineyard |  |
| Land use changed to pasture |  |
| Land use changed to semi-natural system |  |
| Land use changed to tilled vineyard |  |
| Presence of natural habitat |  |
| Presence of oak woodland |  |
| Presence of other agricultural areas |  |
| Presence of remnant vegetation patches |  |
| Presence of riparian zones |  |
| Presence of semi-natural habitat |  |
| Presence of shrub |  |
| Presence of vineyards |  |
| Presence of wildlands |  |
| Presence of woodlands |  |
| Proportion of vineyard |  |
| Presence of wetlands |  |
| Presence of agroecosystem traditional traits | Local scale habitat conditions |
| Forage availability |  |
| Presence of grass cover in specific vineyard areas |  |
| Grass height |  |
| Presence of habitat for natural enemies |  |
| Habitat heterogeneity |  |
| Presence of flowers strips |  |
| Presence of hedgerows and vegetation strips |  |
| Length of hedgerows |  |
| Presence of native buckwheat strips |  |
| Presence of native habitat |  |
| Presence of ecological infrastructure in vineyards |  |
| Presence of nest site for birds |  |
| Presence of oak trees |  |
| Presence solitary trees |  |
| Vineyard block design |  |
| Use of agroforestry practices | Management regime |
| Degree of crop productivity |  |
| Use of irrigation |  |
| Use of nitrogen fertilization |  |
| Use of conventional farming |  |
| Adoption of organic farming |  |
| Presence of organic vineyards |  |
| Copper transport | Nutrients and metals |
| Nitrogen availability |  |
| Presence of nitrates |  |
| Nitrogen deposition |  |
| Nitrogen transport |  |
| Nutrient competition |  |
| Phosphorus transport |  |
| Degree of herbicide use | Pesticide use |
| Degree of pesticide application |  |
| Reduction of fungicide application |  |
| Reduction of pesticide application |  |
| Growing season length | Phenology |
| Length of phenological stages |  |
| Degree of vegetative growth |  |
| Type of canopy management | Vine pruning |
| Use of minimal pruning |  |
| Use of vertical shoot |  |
| Use of local vine varieties | Vine variety |
| Use of optimal vine variety |  |
| Presence of suitable vine varieties |  |
| Type of vine variety |  |
| Use of soil amendment | Vineyard soil |
| Soil depth |  |
| Soil erosion |  |
| Soil organic carbon |  |
| Soil respiration |  |
| Soil structure |  |
| Soil water availability |  |
| Soil water content |  |
| Degree of evapotranspiration | Water availability |
| Water availability |  |
| Water evaporation |  |

**Table S6**: classes used to define the disciplines of the articles and links in our review. We studied the disciplines that considered ecosystem conditions and services based on the research areas defined for the publishing journals included in our sample. We did this analysing the disciplines defined for each article, that were also used to classify the single links between ecosystem services and ecosystem conditions found in the paper. If an article was assigned more disciplines, we considered all of them. To select the disciplines’ classes, we adapted the classification used by Scopus. We did this after checking the consistency between the disciplines assigned by Scopus and Web of Science to the publishing journals included in this review.

| Disciplines |
| --- |
| Agricultural and Biological Sciences |
| Biochemistry, Genetics and Molecular Biology |
| Business, Management and Accounting |
| Earth and Planetary Sciences |
| Economics, Econometrics and Finance |
| Energy |
| Environmental Sciences |
| Neuroscience |
| Medicine |
| Social Sciences |
| Chemistry |
| Materials Sciences |
| Engineering |
| Multidisciplinary |

**Table S7**: representation of the share (%) of how much the relationship between ecosystem condition, ecosystem service or climate change variables were studied in: (a) figure 4a, (b) figure 4b and (c) figure 4c.
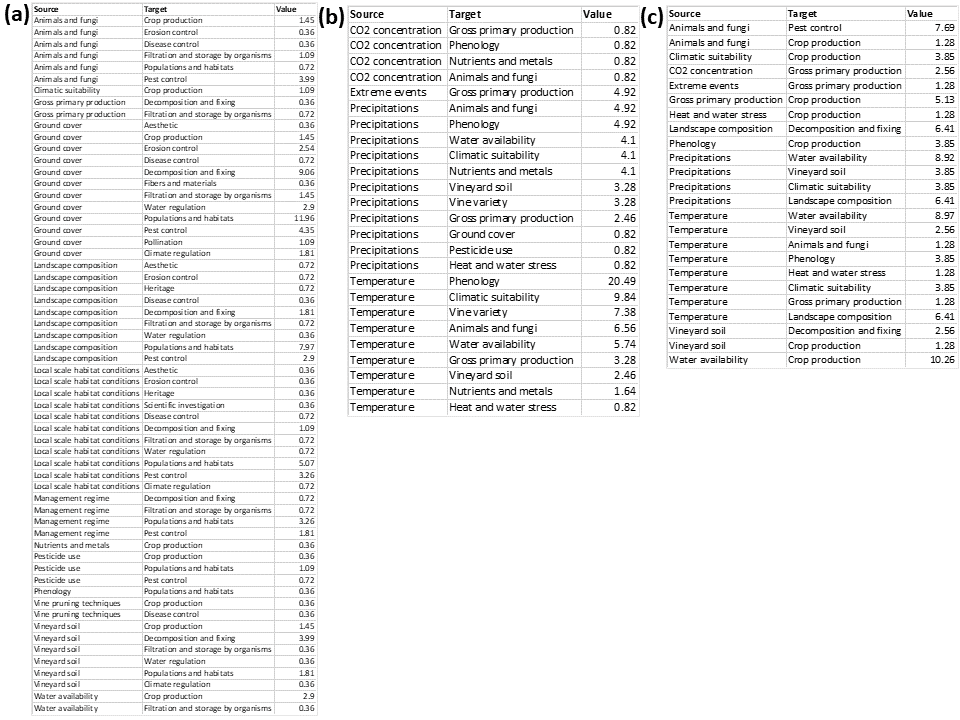


## **Figures**

**
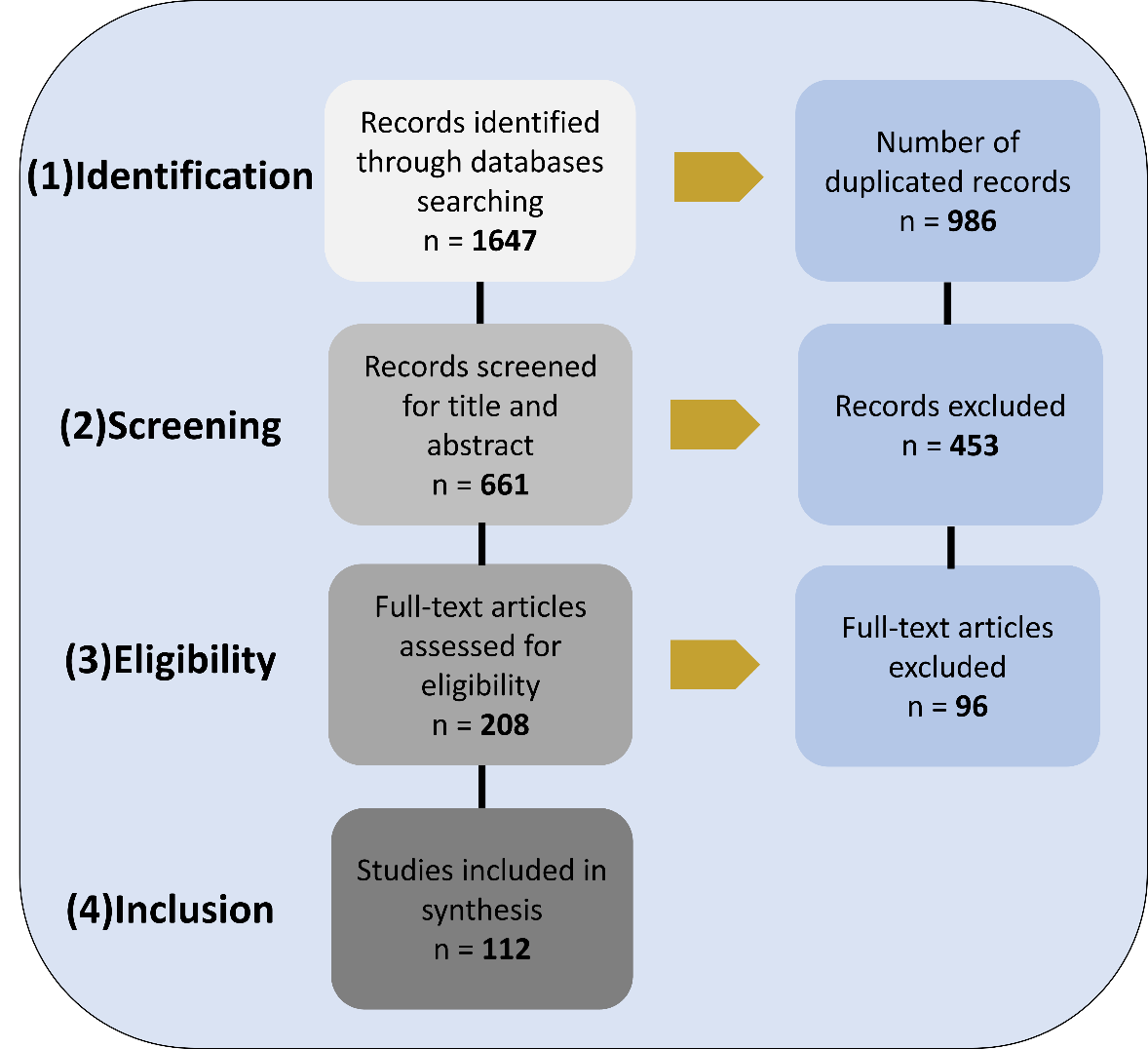
**

**Figure S1**: flow diagram representing the use of the preferred reporting items for systematic reviews and meta-analyses (adapted from Moher et al. (2009), CC BY 4.0) for the selection of the peer-reviewed literature in this review. For each step, we report the number of papers analysed and those excluded. We excluded from our review duplicated papers and papers not matching with our inclusion criteria (table S3).


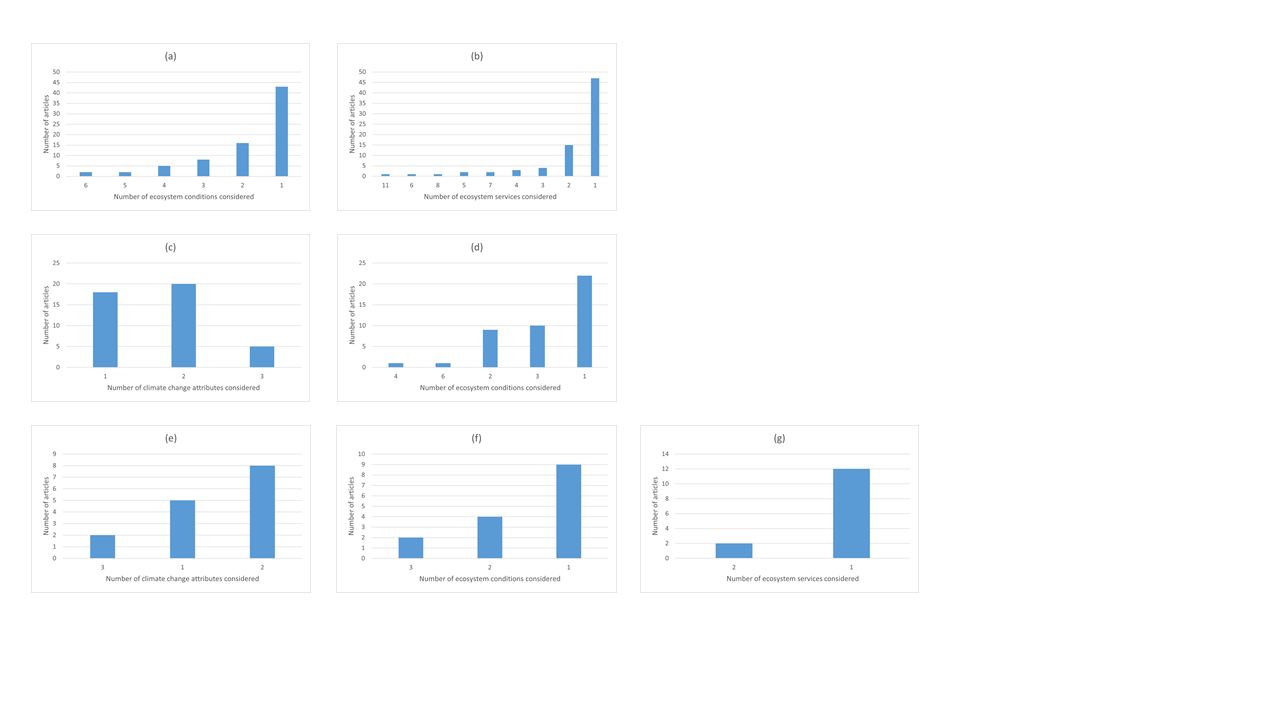


**Figure S2**: frequency in the consideration of climate change attributes, ecosystem conditions and ecosystem services in articles that included: (a,b) *ecosystem conditions*🡪*ecosystem services* links; (c,d) *climate change*🡪*ecosystem conditions* links; (e,f,g) *climate change*🡪*ecosystem conditions*🡪*ecosystem services links*.

**Figure S3:** temporal perspective on the disciplines of the papers of our sample.

**
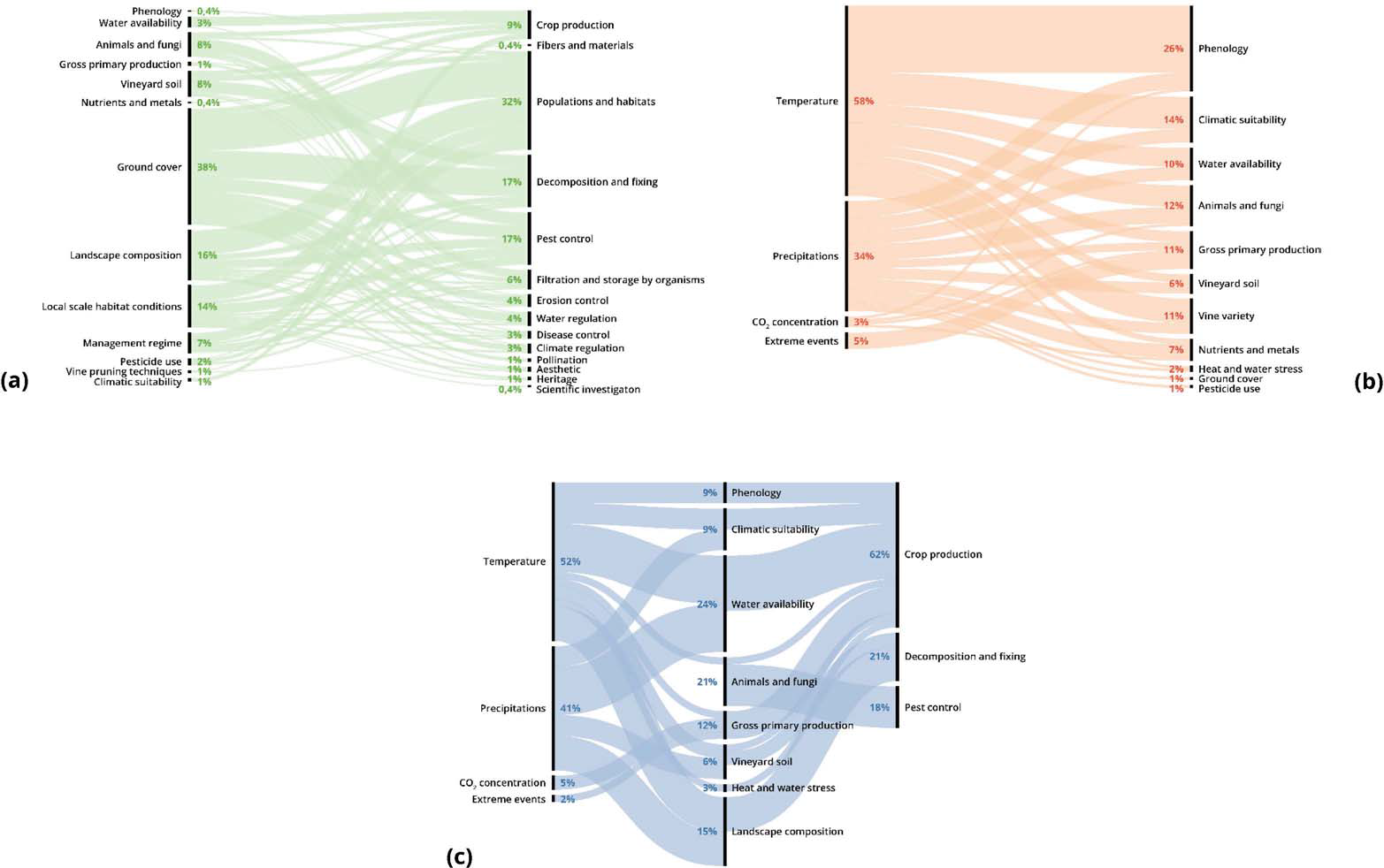
**

**Figure S4:** Sankey diagrams representing: (a) the *ecosystem conditions* 🡪 *ecosystem services* links; (b) *climate change* 🡪 *ecosystem conditions* links; (c) *climate change* 🡪 *ecosystem conditions* 🡪 *ecosystem services* links retrieved in our review. The thickness of the lines is proportional to the total number of links. The percentages in the diagram represent the share of how much the single links’ components and their relationships were studied (see also supplementary table S7). Percentages have been rounded and may not equal 100%.

## **Papers**

List of the 112 articles analysed in this study.

1.Delpuech, X. & Metay, A., 2018. Adapting cover crop soil coverage to soil depth to limit competition for water in a Mediterranean vineyard. Eur. J. Agron. 97, 60–69.

2.Cameron, W. et al., 2019. Advancement of grape maturity: comparison between contrasting cultivars and regions. Aust. J. Grape Wine R. 26, 53–67.

3.Leeuwen, C. van et al., 2019. An Update on the Impact of Climate Change in Viticulture and Potential Adaptations. Agron. 9, 514.

4.Gaitán-Cremaschi, D., Palomo, I., Molina, S. B., Groot, R. D. & Gómez-Baggethun, E., 2017. Applicability of economic instruments for protecting ecosystem services from cultural agrarian landscapes in Doñana, SW Spain. Land Use Policy. 61, 185–195.

5.Muñoz-Rojas, M., Doro, L., Ledda, L. & Francaviglia, R., 2015. Application of CarboSOIL model to predict the effects of climate change on soil organic carbon stocks in agro-silvo-pastoral Mediterranean management systems. Agric. Ecosyst. Environ. 202, 8–16.

6.Crescimanno, G., Morga, F. & Ventrella, D., 2012. Application of the swap model to predict impact of climate change on soil water balance in a Sicilian vineyard. Ital. J. Agron. 7, 17.

7.Trouvelot, S. et al., 2015. Arbuscular mycorrhiza symbiosis in viticulture: a review. Agron. Sustain. Dev. 35, 1449–1467.

8.Winkler, K. J., Viers, J. H. & Nicholas, K. A., 2017. Assessing Ecosystem Services and Multifunctionality for Vineyard Systems. Front. Environ. Sci. 5, 15.

9.Biasi, R., Brunori, E., Ferrara, C. & Salvati, L., 2019. Assessing Impacts of Climate Change on Phenology and Quality Traits of Vitis vinifera L.: The Contribution of Local Knowledge. Plants. 8, 12.

10.Williams, J. N. et al., 2011. Assessment of carbon in woody plants and soil across a vineyard-woodland landscape. Carbon Balance Manag. 6, 11.

11.Barbaro, L. et al., 2016. Avian pest control in vineyards is driven by interactions between bird functional diversity and landscape heterogeneity. J. Appl. Ecol. 54, 500–508.

12.Assandri, G., Bogliani, G., Pedrini, P. & Brambilla, M., 2018. Beautiful agricultural landscapes promote cultural ecosystem services and biodiversity conservation. Agric. Ecosyst. Environ. 256, 200–210.

13.Genesio, L., Miglietta, F., Baronti, S. & Vaccari, F. P., 2015. Biochar increases vineyard productivity without affecting grape quality: Results from a four years field experiment in Tuscany. Agric. Ecosyst. Environ. 201, 20–25.

14.Jiménez-García, L., García-Martínez, Y. G., Marco-Mancebón, V., Pérez, I. & Jiménez-García, D., 2019. Biodiversity analysis of natural arthropods enemies in vineyard agroecosystems in La Rioja, Spain. J. Asia-pac. Entomol. 22, 308–315.

15.Thiéry, D. et al., 2018. Biological protection against grape berry moths. A review. Agron. Sustain. Dev. 38, 15.

16.Hoffmann, C. et al., 2017. Can Flowering Greencover Crops Promote Biological Control in German Vineyards? Insects 8, 121.

17.Francaviglia, R. et al., 2012. Changes in soil organic carbon and climate change – Application of the RothC model in agro-silvo-pastoral Mediterranean systems. Agr. Syst. 112, 48–54.

18.Schultz, H. R., 2000. Climate change and viticulture: A European perspective on climatology, carbon dioxide and UV-B effects. Aust. J. Grape Wine. R 6, 2–12.

19.Fernandez, L., Seixas, F., Oliveira, P., Leitao, S. & Moura, J., 2012. Climate change impacts on nitrogen in a hydrographical basin in the north-east of Portugal. Fresenius Environ. Bull.

20.Fraga, H., Pinto, J. G. & Santos, J. A., 2019. Climate change projections for chilling and heat forcing conditions in European vineyards and olive orchards: a multi-model assessment. Clim. Change. 152, 179–193.

21.Lazoglou, G., Anagnostopoulou, C. & Koundouras, S., 2018. Climate change projections for Greek viticulture as simulated by a regional climate model. Theor. Appl. Climatol. 133, 551–567.

22.Roehrdanz, P. R. & Hannah, L., 2016. Climate Change, California Wine, and Wildlife Habitat. J. Wine. Econ. 11, 69–87.

23.Hannah, L. et al., 2013. Climate change, wine, and conservation. Proc. National Acad. Sci. 110, 6907–6912.

24.Fraga, H. et al., 2015. Climatic suitability of Portuguese grapevine varieties and climate change adaptation. Int. J. Climatol. 36, 1–12.

25.Brambilla, M., Ilahiane, L., Assandri, G., Ronchi, S. & Bogliani, G., 2017. Combining habitat requirements of endemic bird species and other ecosystem services may synergistically enhance conservation efforts. Sci. Total Environ. 586, 206–214.

26.Donkó, Á. et al., 2017. Comparison of species-rich cover crop mixtures in the Tokaj wine region (Hungary). Org. Agric. 7, 133–139.

27.Briche, E., Beltrando, G., Somot, S. & Quenol, H., 2014. Critical analysis of simulated daily temperature data from the ARPEGE-climate model: application to climate change in the Champagne wine-producing region. Clim. Change. 123, 241–254.

28.Orlandini, S., Stefano, V. di, Lucchesini, P., Puglisi, A. & Bartolini, G., 2009. Current trends of agroclimatic indices applied to grapevine in Tuscany (Central Italy). IDŐJÁRÁS.

29.Kolb, S., Uzman, D., Leyer, I., Reineke, A. & Entling, M. H., 2020. Differential effects of semi-natural habitats and organic management on spiders in viticultural landscapes. Agric. Ecosyst. Environ. 287, 106695.

30.Judt et al., 2019. Diverging Effects of Landscape Factors and Inter-Row Management on the Abundance of Beneficial and Herbivorous Arthropods in Andalusian Vineyards (Spain). Insects. 10, 320.

31.Hogg, B. N. & Daane, K. M., 2011. Diversity and invasion within a predator community: impacts on herbivore suppression: Exotic spider impacts in a crop system. J. Appl. Ecol. 48, 453–461.

32.Rasran, L., Diener, A., Pachinger, B. & Bernhardt, K.G., 2018. Diversity of Flower Visiting Insects in Dry Grasslands and Vineyards Close to the City of Vienna with Special Focus on Wild Bees. Sociobiology. 65, 603–61.

33.Hogg, B. N. & Daane, K. M., 2011. Ecosystem services in the face of invasion: the persistence of native and nonnative spiders in an agricultural landscape. Ecol. Appl. 21, 565–576.

34.Ungureanu, G. et al., 2017. Effect of Climate Change on Pedological Modifications and Soil Aridity Process in Vineyards. Revista de Chimie.

35.Torres, R. L., Lloreda, M. D. L. F., Gonzalez, P. J., García-Gutierrez, J. R. L. & Trujillo, P. B., 2018. Effect of soil management strategies on the characteristics of the grapevine root system in irrigated vineyards under semi-arid conditions. Aust. J. Grape Wine R. 24, 439–449.

36.Castañeda, L. E., Miura, T., Sánchez, R. & Barbosa, O., 2018. Effects of agricultural management on phyllosphere fungal diversity in vineyards and the association with adjacent native forests. Peerj. 6, e5715.

37.Tixier, M.-S., Arnaud, A., Douin, M. & Kreiter, S., 2015. Effects of agroforestry on Phytoseiidae communities (Acari: Mesostigmata) in vineyards. A synthesis of a 10-year period of observations. Acarologia. 55, 361–375.

38.Eldon, J. & Gershenson, A., 2015. Effects of Cultivation and Alternative Vineyard Management Practices on Soil Carbon Storage in Diverse Mediterranean Landscapes: A Review of the Literature. Agroecol. Sust. Food. 39, 150128131234005.

39.Nereu, M., Heleno, R. H., Lopez-Núñez, F., Agostinho, M. & Ramos, J. A., 2018. Effects of native biodiversity on grape loss of four castes: testing the biotic resistance hypothesis. Web. Ecol. 18, 15–27.

40.Ramos, M. C. & Martínez-Casasnovas, J. A., 2010. Effects of precipitation patterns and temperature trends on soil water available for vineyards in a Mediterranean climate area. Agr. Water Manage. 97, 1495–1505.

41.Costantini, E. A. C. et al., 2018. Effects of soil erosion on agro-ecosystem services and soil functions: A multidisciplinary study in nineteen organically farmed European and Turkish vineyards. J. Environ. Manage. 223, 614–624.

42.Winter, S. et al., 2018. Effects of vegetation management intensity on biodiversity and ecosystem services in vineyards: A meta-analysis. J. Appl. Ecol. 55, 2484–2495.

43.Fiera, C. et al., 2020. Effects of vineyard inter-row management on the diversity and abundance of plants and surface-dwelling invertebrates in Central Romania. J. Insect Conserv. 24, 175–185.

44.Jacometti, M. A., Wratten, S. D. & Walter, M., 2011. Enhancing ecosystem services in vineyards: using cover crops to decrease botrytis bunch rot severity. Int. J. Agric. Sustain. 5, 305–314.

45.Machar, I. et al., 2017. Environmental Modelling of Climate Change Impact on Grapevines: Case Study from the Czech Republic. Pol. J. Environ. Stud. 26, 1927–1934.

46.Miglécz, T. et al., 2015. Establishment of three cover crop mixtures in vineyards. Sci. Hortic.-amsterdam. 197, 117–123.

47.Paiola, A. et al., 2019. Exploring the potential of vineyards for biodiversity conservation and delivery of biodiversity-mediated ecosystem services: A global-scale systematic review. Sci. Total Environ. 706, 135839.

48.Schultz, H. R., 2016. Global Climate Change, Sustainability, and Some Challenges for Grape and Wine Production*. J. Wine Econ. 11, 181–200.

49.Rusch, A., Delbac, L. & Thiéry, D., 2017. Grape moth density in Bordeaux vineyards depends on local habitat management despite effects of landscape heterogeneity on their biological control. J. Appl. Ecol. 54, 1794–1803.

50.Bernardo, S., Dinis, L.-T., Machado, N. & Moutinho-Pereira, J., 2018. Grapevine abiotic stress assessment and search for sustainable adaptation strategies in Mediterranean-like climates. A review. Agron. Sustain Dev. 38, 66.

51.Cola, G., Failla, O., Maghradze, D., Megrelidze, L. & Mariani, L., 2017. Grapevine phenology and climate change in Georgia. Int. J. Biometeorol. 61, 761–773.

52.Sáenz-Romo, M. G. et al., 2019. Ground cover management in a Mediterranean vineyard: Impact on insect abundance and diversity. Agric. Ecosyst. Environ. 283, 106571.

53.Kelly, R. M., Kitzes, J., Wilson, H. & Merenlender, A., 2016. Habitat diversity promotes bat activity in a vineyard landscape. Agric. Ecosyst. Environ. 223, 175–181.

54.Burgio, G. et al., 2016. Habitat management of organic vineyard in Northern Italy: the role of cover plants management on arthropod functional biodiversity. B. Entomol. Res. 106, 759–768.

55.Nesbitt, A., Kemp, B., Steele, C., Lovett, A. & Dorling, S., 2016. Impact of recent climate change and weather variability on the viability of UK viticulture - combining weather and climate records with producers’ perspectives: Climate and weather impacts on UK viticulture. Aust. J. Grape Wine R. 22, 324–335.

56.Serpa, D., Nunes, J. P., Keizer, J. J. & Abrantes, N., 2017. Impacts of climate and land use changes on the water quality of a small Mediterranean catchment with intensive viticulture. Environ. Pollut. 224, 454–465.

57.Fuhrer, J., Smith, P. & Gobiet, A., 2014. Implications of climate change scenarios for agriculture in alpine regions — A case study in the Swiss Rhone catchment. Sci. Total Environ. 493, 1232–1241.

58.Pedro, A. R.-S. et al., 2018. Influence of agricultural management on bat activity and species richness in vineyards of central Chile. J. Mammal. 99, 1495–1502..

59.Rusch, A., Binet, D., Delbac, L. & Thiéry, D., 2016. Local and landscape effects of agricultural intensification on Carabid community structure and weed seed predation in a perennial cropping system. Landscape Ecol. 31, 2163–2174.

60.Capó-Bauçà, S., Marqués, A., Llopis-Vidal, N., Bota, J. & Baraza, E., 2019. Long-term establishment of natural green cover provides agroecosystem services by improving soil quality in a Mediterranean vineyard. Ecol. Eng. 127, 285–291.

61.Garcia, L. et al., 2018. Management of service crops for the provision of ecosystem services in vineyards: A review. Agric. Ecosyst. Environ. 251, 158–170.

62.Fiedler, A. K., Landis, D. A. & Wratten, S. D., 2008. Maximizing ecosystem services from conservation biological control: The role of habitat management. Biol. Control. 45, 254–271.

63.Pennington, T., Kraus, C., Alakina, E., Entling, M. & Hoffmann, C., 2017. Minimal Pruning and Reduced Plant Protection Promote Predatory Mites in Grapevine. Insects. 8, 86.

64.Wolff, M. W., Alsina, M. M., Stockert, C. M., Khalsa, S. D. S. & Smart, D. R., 2018. Minimum tillage of a cover crop lowers net GWP and sequesters soil carbon in a California vineyard. Soil Tillage Res. 175, 244–254.

65.Fraga, H., Atauri, I. G. de C., Malheiro, A. C. & Santos, J. A., 2016. Modelling climate change impacts on viticultural yield, phenology and stress conditions in Europe. Global Change Biol. 22, 3774–3788.

66.Leolini, L. et al., 2019. Modelling sugar and acid content in Sangiovese grapes under future climates: an Italian case study. Clim. Res. 78, 211–224.

67.Leeuwen, C. van & Destrac-Irvine, A., 2017. Modified grape composition under climate change conditions requires adaptations in the vineyard. Oeno One. 51, 147–154.

68.Nistor, E. et al., 2018. N2O, CO2, Production, and C Sequestration in Vineyards: a Review. Water Air Soil Pollut. 229, 299.

69.Eckert, M., Mathulwe, L. L., Gaigher, R., Merwe, L. J. der & Pryke, J. S., 2020. Native cover crops enhance arthropod diversity in vineyards of the Cape Floristic Region. J. Insect Conserv. 24, 133–149.

70.Daane, K. M., Hogg, B. N., Wilson, H. & Yokota, G. Y., 2018. Native grass ground covers provide multiple ecosystem services in Californian vineyards. J. Appl. Ecol. 55, 2473–2483.

71.Rusch, A., Delbac, L., Muneret, L. & Thiéry, D., 2015. Organic farming and host density affect parasitism rates of tortricid moths in vineyards. Agric. Ecosyst. Environ. 214, 46–53.

72.Muneret, L., Auriol, A., Thiéry, D. & Rusch, A., 2018. Organic farming at local and landscape scales fosters biological pest control in vineyards. Ecol. Appl. 29, e01818.

73.Muneret, L. et al., 2019. Organic farming expansion drives natural enemy abundance but not diversity in vineyard‐dominated landscapes. Ecol. Evol. 9, 13532–13542.

74.Rollan, À., Hernández-Matías, A. & Real, J., 2019. Organic farming favours bird communities and their resilience to climate change in Mediterranean vineyards. Agric. Ecosyst. Environ. 269, 107–115.

75.Caprio, E., Nervo, B., Isaia, M., Allegro, G. & Rolando, A., 2015. Organic versus conventional systems in viticulture: Comparative effects on spiders and carabids in vineyards and adjacent forests. Agr. Syst. 136, 61–69.

76.Ramos, M. C., Jones, G. V. & Yuste, J., 2018. Phenology of Tempranillo and Cabernet-Sauvignon varieties cultivated in the Ribera del Duero DO: observed variability and predictions under climate change scenarios. Oeno One. 52.

77.Shields, M. W., Tompkins, J.-M., Saville, D. J., Meurk, C. D. & Wratten, S., 2016. Potential ecosystem service delivery by endemic plants in New Zealand vineyards: successes and prospects. Peerj. 4, e2042.

78.Carlo, P. D., Aruffo, E. & Brune, W. H., 2019. Precipitation intensity under a warming climate is threatening some Italian premium wines. Sci. Total Environ. 685, 508–513.

79.Retallack, M. J., Thomson, L. J. & Keller, M. A., 2019. Predatory arthropods associated with potential native insectary plants for Australian vineyards. Aust. J. Grape Wine R. 25, 233–242.

80.Ramos, M. C., 2017. Projection of phenology response to climate change in rainfed vineyards in north-east Spain. Agr. For. Meteorol. 247, 104–115.

81.Gutierrez, A. P., Daane, K. M., Ponti, L., Walton, V. M. & Ellis, C. K., 2008. Prospective evaluation of the biological control of vine mealybug: refuge effects and climate. J. Appl. Ecol. 45, 524–536.

82.Rayne, S. & Forest, K., 2016. Rapidly changing climatic conditions for wine grape growing in the Okanagan Valley region of British Columbia, Canada. Sci. Total Environ. 556, 169–178.

83.Holland, T. & Smit, B., 2014. Recent climate change in the Prince Edward County winegrowing region, Ontario, Canada: implications for adaptation in a fledgling wine industry. Reg. Environ. Change. 14, 1109–1121.

84.Pennington, T., Reiff, J. M., Theiss, K., Entling, M. H. & Hoffmann, C., 2018. Reduced fungicide applications improve insect pest control in grapevine. Biocontrol. 63, 687–695.

85.Polyakov, A. Y., Weller, T. J. & Tietje, W. D., 2019. Remnant trees increase bat activity and facilitate the use of vineyards by edge-space bats. Agric. Ecosyst. Environ. 281, 56–63.

86.Ruml, M., Kora, N., Vujadinović, M., Vuković, A. & Ivanišević, D., 2016. Response of grapevine phenology to recent temperature change and variability in the wine-producing area of Sremski Karlovci, Serbia. J. Agric. Sci. 154, 186–206.

87.Kratschmer, S. et al., 2019. Response of wild bee diversity, abundance, and functional traits to vineyard inter‐row management intensity and landscape diversity across Europe. Ecol. Evol. 9, 4103–4115.

88.Pedro, A. R.-S. et al., 2019. Responses of aerial insectivorous bats to landscape composition and heterogeneity in organic vineyards. Agric. Ecosyst. Environ. 277, 74–82.

89.Martinez-Harms, M. J. et al., 2017. Scenarios for land use and ecosystem services under global change. Ecosyst. Serv. 25, 56–68.

90.Martínez-Lüscher, J. et al., 2016. Sensitivity of Grapevine Phenology to Water Availability, Temperature and CO2 Concentration. Front. Environ. Sci. 4, 48.

91.Novara, A. et al., 2020. Soil Carbon Budget Account for the Sustainability Improvement of a Mediterranean Vineyard Area. Agronomy 10, 336.

92.Costantini, E. et al., 2018. Soil functions and effects of soil erosion. First evidences from the resolve project. Environ. Quality.

93.Gristina, L. et al., 2019. Soil organic carbon stocks under recommended management practices in different soils of semiarid vineyards. Land Degrad Dev.

94.Ramos, M. C., 2006. Soil water content and yield variability in vineyards of Mediterranean northeastern Spain affected by mechanization and climate variability. Hydrol. Process. 20, 2271–2283.

95.Neethling, E., Barbeau, G., Coulon-Leroy, C. & Quénol, H., 2019. Spatial complexity and temporal dynamics in viticulture: A review of climate-driven scales. Agr. For. Meteorol. 276–277, 107618.

96.Novikova, L. Y. & Naumova, L. G., 2019. Structuring ampelographic collections by phenotypic characteristics and comparing the reaction of grape varieties to climate change. Vavilovskij ž. Genet. Sel. 23, 772–779.

97.Wilson, H. et al., 2017. Summer Flowering Cover Crops Support Wild Bees in Vineyards. Environ. Entomol. 47, 63–69.

98.Novara, A., Cerdà, A. & Gristina, L., 2018. Sustainable vineyard floor management: an equilibrium between water consumption and soil conservation. Curr. Opin. Environ Sci. Heal. 5, 33–37.

99.Brunori, E., Farina, R. & Biasi, R., 2016. Sustainable viticulture: The carbon-sink function of the vineyard agro-ecosystem. Agric. Ecosyst. Environ. 223, 10–21.

100.Rosas‐Ramos, N., Baños‐Picón, L., Tormos, J. & Asís, J. D., 2019. The complementarity between ecological infrastructure types benefits natural enemies and pollinators in a Mediterranean vineyard agroecosystem. Ann. Appl. Biol. 175, 193–201.

101.Sommaggio, D., Peretti, E. & Burgio, G., 2018. The effect of cover plants management on soil invertebrate fauna in vineyard in Northern Italy. Biocontrol. 63, 795–806.

102.Gonçalves, F. et al., 2018. The functional agrobiodiversity in the Douro demarcated region viticulture: utopia or reality? Arthropods as a case-study – A review. Ciência E Técnica Vitivinícola 34, 102–114.

103.Sgubin, G., Swingedouw, D., Cortázar-Atauri, I. G. de, Ollat, N. & Leeuwen, C. van, 2019. The Impact of Possible Decadal-Scale Cold Waves on Viticulture over Europe in a Context of Global Warming. Agronomy. 9, 397.

104.Jones, N. K., 2012. The influence of recent climate change on wine regions in Quebec, Canada. J. Wine Res. 23, 103–113.

105.Sottini, V. A. et al., 2019. The use of crowdsourced geographic information for spatial evaluation of cultural ecosystem services in the agricultural landscape: the case of chianti classico (Italy). New Medit. 18, 105–118.

106.Lereboullet, A.-L., Beltrando, G., Bardsley, D. K. & Rouvellac, E., 2013. The viticultural system and climate change: coping with long-term trends in temperature and rainfall in Roussillon, France. Reg. Environ. Change. 14, 1951–1966.

107.Kratschmer, S. et al., 2018. Tillage intensity or landscape features: What matters most for wild bee diversity in vineyards? Agric. Ecosyst. Environ. 266, 142–152.

108.Garcia, L. et al., 2019. Trait-based approach for agroecology: contribution of service crop root traits to explain soil aggregate stability in vineyards. Plant Soil. 435, 1–14.

109.Fraga, H. et al., 2014. Very high resolution bioclimatic zoning of Portuguese wine regions: present and future scenarios. Reg. Environ. Change. 14, 295–306.

110.Viers, J. H. et al., 2013. Vinecology: pairing wine with nature: Vinecology. Conserv. Lett. 6.

111.Jones, J. E., Kerslake, F. L., Close, D. C. & Dambergs, R. G., 2014. Viticulture for Sparkling Wine Production: A Review. Am. J. Enol. Viticult. 65, 407–416.

112.Castex, V., Tejeda, E. M. & Beniston, M., 2015. Water availability, use and governance in the wine producing region of Mendoza, Argentina. Environ. Sci. Policy. 48, 1–8.

**References**

Haines-Young R, Potschin MB, (2018). Revision of the Common International Classification for Ecosystem Services (CICES V5.1): A Policy Brief. One Ecosyst 3:e27108. https://doi.org/10.3897/oneeco.3.e27108.

Maes J, Teller A, Erhard M, Grizzetti B, Barredo JI, Paracchini ML, Condé S, Somma F, Orgiazzi A, Jones A, Zulian G, Vallecilo S, Petersen JE, Marquardt D, Kovacevic V, Malak DA, Marin AI, Czúcz B, Mauri A, … Werner B (2018). Mapping and Assessment of Ecosystems and their Services. An analytical framework for mapping and assessment of ecosystem conditions in EU. Publications office of the European Union, Luxembourg.

Moher D, Liberati A, Tetzlaff J, Altman DG, Group TP (2009) Preferred Reporting Items for Systematic Reviews and Meta-Analyses: The PRISMA Statement. PLoS Med 6(7):e1000097. https://doi.org/10.1371/journal.pmed.1000097.

Winkler KJ, Viers JH, Nicholas KA (2017). Assessing Ecosystem Services and Multifunctionality for Vineyard Systems. Front Environ Sci 5:15. https://doi.org/10.3389/fenvs.2017.00015.
